# Component Distribution, Shear-Flow Behavior, and Sol–Gel Transition in Mixed Dispersions of Casein Micelles and Serum Proteins

**DOI:** 10.3390/foods13213480

**Published:** 2024-10-30

**Authors:** Hossein Gholamian, Maksym Loginov, Marie-Hélène Famelart, Florence Rousseau, Fabienne Garnier-Lambrouin, Geneviève Gésan-Guiziou

**Affiliations:** UMR 1253 Science et Technologie du Lait et de L’Oeuf (STLO), INRAE, Institut Agro, 35000 Rennes, France; hossein.gholamian@inrae.fr (H.G.); marie-helene.famelart@inrae.fr (M.-H.F.); florence.rousseau@inrae.fr (F.R.); fabienne.lambrouin@inrae.fr (F.G.-L.); genevieve.gesan-guiziou@inrae.fr (G.G.-G.)

**Keywords:** casein micelle, whey protein, shear rheology, sol–gel transition, viscosity, voluminosity

## Abstract

The shear flow and solid–liquid transition of mixed milk protein dispersions with varying concentrations of casein micelles (CMs) and serum proteins (SPs) are integral to key dairy processing operations, including microfiltration, ultrafiltration, diafiltration, and concentration–evaporation. However, the rheological behavior of these dispersions has not been sufficiently studied. In the present work, dispersions of CMs and SPs with total protein weight fractions (*ω_PR_*) of 0.021–0.28 and SP to total protein weight ratios (*R_SP_*) of 0.066–0.214 and 1 were prepared by dispersing the respective protein isolates in the permeate from skim milk ultrafiltration and then further concentrated via osmotic compression. The partition of SPs between the CMs and the dispersion medium was assessed by measuring the dry matter content and viscosity of the dispersion medium after separating it from the CMs via ultracentrifugation. The rheological properties were studied at 20 °C via shear rheometry, and the sol–gel transition was characterized via oscillatory measurements. No absorption of SPs by CMs was observed in dispersions with *ω_PR_* = 0.083–0.126, regardless of the *R_SP_*. For dispersions of SPs with *ω_PR_* ≤ 0.21, as well as the dispersion medium of mixed dispersions with *ω_PR_* = 0.083–0.126, the high shear- rate-limiting viscosity was described using Lee’s equation with an SP voluminosity (*v_SP_*) of 2.09 mL·g^−1^. For the mixed dispersions with a CM volume fraction of *φ_CM_* ≤ 0.37, the relative high shear-rate-limiting viscosity was described using Lee’s equation with a CM voluminosity (*v_CM_*) of 4.15 mL·g^−1^ and a *v_SP_* of 2.09 mL·g^−1^, regardless of the *R_SP_*. For the mixed dispersions with *φ_CM_* > 0.55, the relative viscosity increased significantly with an increasing *R_SP_* (this was explained by an increase in repulsion between CMs). However, the sol–gel transition was independent of the *R_SP_* and was observed at *φ_CM_* ≈ 0.65.

## 1. Introduction

Characterizing the rheological behavior of mixed milk protein dispersions can be helpful for optimizing key dairy processing operations related to shear flow and liquid-to-solid transition, such as concentration–evaporation, microfiltration, ultrafiltration, and diafiltration. However, this is a complex task because, in addition to the operating parameters (shear stress and temperature), this behavior depends on the composition of processed dispersions and interactions between their constituents.

These dispersions (i.e., skim milk and its derivatives—retentates and concentrates) can be described as a system of nanoparticles (casein micelles (CMs)) in an aqueous dispersion medium (serum (dm)).

CMs are natural aggregates of *α_S_*_1_-, *α_S_*_2_-, *β*-, and *κ*-caseins (CNs) held together via electrostatic and hydrophobic interactions and additionally connected by “bridges” of Ca^2+^ and calcium phosphate nanocrystals. CMs are swollen in an aqueous medium. Swollen CMs have a nearly spherical shape, a broad size distribution, and a mean diameter of 150–200 nm. They have a core–shell structure, with a soft and porous (and thus compressible and deformable) core composed of *α_S_*_1_-, *α_S_*_2_-, and *β*-CNs and a shell composed of the hydrophilic parts of *κ*-CNs, with a thickness of ca. 5 nm. This shell ensures the stability of CMs against aggregation. The extent of CM swelling is conventionally characterized by CM voluminosity (*v_CM_*)

(1) $$v_{CM}=V_{CM}/m_{mCN}$$

where *V_CM_* is the volume of swollen CMs, and *m_mCN_* is the weight of micellar CNs (mCNs) (that are CNs aggregated into CMs). It must be noted that the value of *V_CM_* (and thus *v_CM_*) can vary depending on the property used to define it (e.g., the quantity of water or serum absorbed, the effective hydrodynamic radius of CMs, etc.). Moreover, *v_CM_* depends on the factors that influence the CN-CN and CN-dm interactions, e.g., temperature, pH, and serum composition.

Serum (dm) is an aqueous solution of macromolecules (serum proteins (SPs)) and low-molecular-weight organic and inorganic compounds. The most abundant SPs are the whey proteins (WPs) β-lactoglobulin (molecular weight = 18.3 kDa (monomer) and 36.6 kDa (dimer)) and *α*-lactalbumin (molecular weight = 14.2 kDa). Minor WPs include immunoglobulins, bovine serum albumin, and lactoferrin. CMs and dm co-exist in a dynamic equilibrium. Decreasing the temperature (e.g., during the cold storage of milk before filtration) or ionic strength of the dm (e.g., after diluting milk retentate with water during diafiltration) can weaken the structure of CMs and cause some of the CN molecules to be extracted from CMs in the dm. These individual CN molecules present in the dm of mixed milk protein dispersions (i.e., serum CNs (sCNs)) are thus quantified as SPs along with WPs. In this regard, it is worth mentioning that Equation (1) is sometimes applied with *m_CN_* instead of *m_mCN_*. This approach is suitable for the empirical characterization of CM dispersions; however, it is not appropriate for characterizing CMs themselves.

Organic low-molecular-weight compounds of serum (o) include lactose (the most abundant soluble component of skim milk serum, concentration ≈ 0.05 g·mL^−1^), peptides, and organic acids. Inorganic (mineral) compounds of serum (eMN) include K^+^, Na^+^, Ca^2+^, Mg^+2,^ and their salts (mainly phosphates).

Given the size of CMs and SPs (several nm vs. ca. 150–200 nm), it is reasonable to expect that, along with the total protein weight fraction (*ω_PR_*), the relative content of SPs (*R_SP_*) also influences the rheological properties of mixed milk protein dispersions:

(2) $$\omega_{PR}=\omega_{SP}+\omega_{mCN}$$

(3) $$R_{SP}=\omega_{SP}/\omega_{PR}$$

where *ω_SP_* and *ω_mCNs_* are the weight fractions of SPs and mCNs in the dispersion, respectively.

Total concentrations of CNs, β-lactoglobulin, and *α*-lactalbumin in skim milk are ca. 0.02–0.03, 0.003–0.004, and 0.001–0.0015 (g·mL^−1^), respectively. Thus, the *R_SP_* of skim milk is typically ≈ 0.2. Because SPs are much smaller than CMs, their concentrations vary differently across dairy processing: microfiltration and dia-microfiltration will reduce the *R_SP_*, even when the concentration of CMs in the retentate remains constant, while concentration via ultrafiltration or evaporation increases the total protein concentration without changing the *R_SP_*.

It is important to note that determining *ω_SP_* from the total concentration of WPs in mixed dispersions of SPs and CMs is not trivial: the literature on the distribution of WPs between the dm and swollen CMs appears to be contradictory. For example, Anema et al. [1] did not detect any absorption of WPs by CMs in skim milk, and Ahmadi et al. [2,3] reported that only about 2–5% of the total WPs of skim milk are associated with CMs. However, Peixoto et al. [4] reported that for a wide range *R_SP_* in mixed dispersions of WPs and CMs, WPs were absorbed by CMs with a partition coefficient between CMs and dm of ca. 4–8 (regardless of the CM concentration). This complicates the analysis of how SPs and CMs influence the rheological properties of mixed dispersions.

Early studies on the rheology of mixed dispersions of SPs and CMs were reviewed by [5]. Unfortunately, different sources of milk proteins were used, and different equations, often empirical, were applied within these studies; this makes difficult the comparison and analysis of their results. Thus, we reviewed a selection of more recent studies that provided the most complete and systematic descriptions and analyses of the rheological properties of (1) CM dispersions with a constant low *R_SP_* (0.05–0.10), (2) concentrated milk with a constant high *R_SP_* (0.20–0.25), and (3) mixed milk protein dispersions with a variable *R_SP_*.

### 1.1. Shear-Flow Properties of CM Dispersions with Low R_SP_

Bouchoux et al. [6] studied the shear-flow behavior and the liquid-to-solid transition of CM dispersions with constant low *R_SP_* (ca. 0.05–0.06) at 20 °C. Dispersions with CN concentrations of 0.01–0.40 g·mL^−1^ were prepared by thoroughly mixing micellar casein isolate powder (CNI) in a native dm of CMs (permeate from skim milk ultrafiltration (PUF)) followed by an optional concentration via osmotic compression. They assumed a constant CM voluminosity (*v_CM_* = 4.4 mL·g^−1^) for the data analysis.

They observed that dispersions with a CN concentration < 0.1 g·mL^−1^ (i.e., with a volume fraction of CMs (*φ_CM_*) < 0.44) behaved as Newtonian fluids. When the CN concentration in dispersions exceeded 0.1 g·mL^−1^ (i.e., *φ_CM_* > 0.44), the dispersion displayed shear thinning behavior.

At 0.05 < *φ_CM_* ≤ 0.3, the relative viscosity (calculated under the assumption that the viscosity of the dm (*η_dm_*) is constant regardless of the protein concentration in the system and equals that of the pure solvent, i.e., PUF: *η_dm_* = *η_PUF_*) was well described by the equation of de Kruif for hard spheres [7]:

(4) $$\eta_{rPUF}=1+2.5\varphi +4\varphi^{2}+42\varphi^{3}$$

where *φ* is the volume fraction of particles (here, CMs).

Shear-flow curves for dispersions with casein concentrations of 0.121–0.168 g·mL^−1^ (i.e., *φ_CM_* = 0.53–0.74) were well described by the equation of Krieger and Dougherty, which represents pure hydrodynamic interactions of hard spheres [8]:

(5) $$\frac{\eta -\eta_{\infty }}{\eta_{0}-\eta_{\infty }}={\left(1+\frac{\tau }{\tau_{c}}\right)}^{-1}$$

Here, η is the apparent viscosity of the dispersion, *τ* is the applied shear stress, and *η*_0_, *η*_∞_, and *τ_c_* are fitting parameters: *η*_0_ is the zero-shear-rate viscosity (*η*_0_ = $\underset{\dot{\gamma }\to 0}{\lim}\eta$), *η*_∞_ is the infinite-shear-rate viscosity (*η*_∞_ = $\underset{\dot{\gamma }\to \infty }{\lim}\eta$), and *τ_c_* is the characteristic shear stress (according to Krieger and Dougherty, *τ_c_* depends on the particle size). It must be noted that the values of *τ_c_* obtained by Bouchoux et al. [6] passed through a sharp maximum as the *φ_CM_* increased; this dependence of *τ_c_* on *φ_CM_* was not described theoretically.

The obtained dependencies $\eta_{0}\left(\varphi_{CM}\right)$ and $\eta_{\infty }\left(\varphi_{CM}\right)$ were described by the equation of Quemada for hard spheres [9]:

(6) $$\eta_{rPUF,0/\infty }={\left(1+\frac{\varphi_{CM}}{\varphi_{CMmax,0/\infty }}\right)}^{-2}$$

where $\eta_{rPUF,0/\infty }$ is either the zero (0) or infinite (∞) shear-rate relative viscosity and $\varphi_{CMmax,0/\infty }$ is the maximum packing fraction of CMs, at which the corresponding (low (0) or high (∞)) shear-rate viscosity diverges (becomes infinite). The values assumed for $\varphi_{CMmax,0}$ and $\varphi_{CMmax,\infty }$ were 0.64 and 0.78, respectively; applying Equation (4) greatly underestimated $\eta_{rPUF,0}$ and $\eta_{rPUF,\infty }$ for *φ_CM_* = 0.53–0.74.

Based on the oscillatory measurements, it was concluded that the sol–gel transition occurred in the range of *φ_CM_* = 0.64–0.78. Since $\eta_{rPUF,0}$ and $\eta_{rPUF,\infty }$ did not diverge in this region, as would be expected for a hard spheres system. Bouchoux et al. [6] suggested that, at *φ_CM_* ≥ 0.53, the rheological behavior is influenced by shear-induced deformations of CMs.

More recently, Doudiès et al. [10] performed a similar study for dispersions of CNI in PUF (*R_SP_* ≈ 0.09) at 7 °C and 20 °C, assuming *v_CM,_*_7 *°C*_ = 4.85 mL·g^−1^ [11] and *v_CM,_*_20*°C*_ = 4.10 mL·g^−1^ [11] for the data analysis.

In agreement with the results of Bouchoux et al. [6], dispersions became essentially non-Newtonian when the CN concentrations exceeded 0.1 g·mL^−1^ (i.e., *φ_CM_* > 0.485 and 0.410 at 7 °C and 20 °C, respectively).

Relative viscosity was calculated assuming *η_dm_* = *η_PUF_*; dependencies $\eta_{rPUF}\left(\varphi_{CM}\right)$ were obtained for an arbitrarily chosen shear rate of $\dot{\gamma }$ = 0.1 s^−1^ (which was beyond the zero-shear-rate domain for most concentrated dispersions). The dependencies followed the same trend at 7 °C and 20 °C and were only approximately described by Equation (6), assuming *φ_CMmax_* = 0.71. Oscillatory measurements confirmed that the sol–gel transition occurred at *φ_CM_* = 0.71 regardless of the temperature.

Dahbi et al. [12] studied the rheological properties of dispersions prepared by mixing CNI powder (*R_SP_* = 0.08) into water. It should be noted that these authors were obliged to assume two greatly different values of *v_CM_* (3.4 mL·g^−1^ and 4.4 mL·g^−1^) to obtain comparable results for the two sets of dispersions they prepared.

The dispersions revealed distinct shear thinning for CN concentrations ≥ 0.0684 g·g^−1^ (i.e., *φ_CM_* ≥ 0.23 or 0.30, depending on the assumed value of *v_CM_*); this limiting concentration was much lower than that reported by Bouchoux et al. [6] and Doudiès et al. [10] (i.e., 0.1 g·mL^−1^).

Within the wide range of CN concentrations of 0.0684–0.1789 g·g^−1^ (*φ_CM_* = 0.23–0.61 or 0.30–0.79, depending on the assumed value of *v_CM_*), shear-flow curves were well described by the equation of Cross [13] (which is essentially different from Equation (5), successfully applied by [6]):

(7) $$\frac{\eta -\eta_{\infty }}{\eta_{0}-\eta_{\infty }}={\left(1+{\left(\frac{\dot{\gamma }}{{\dot{\gamma }}_{c}}\right)}^{q}\right)}^{-1}$$

Here, the exponent *q* and the critical shear rate ${\dot{\gamma }}_{c}$ are fitting parameters. The best fit was obtained when *q* = 0.62 (regardless of the CN concentration); unfortunately, the values of ${\dot{\gamma }}_{c}$ and $\eta_{\infty }$ were not provided or discussed.

Zero-shear-rate relative viscosity (*η_r,_*_0_) was calculated using the viscosity of centrifugal ultrafiltrates of dispersions instead of *η_dm_*; the authors did not consider that the obtained values of *η_dm_* can be influenced by the rejection of SPs during the ultrafiltration.

The obtained dependency of *η_r,_*_0_ on *φ_CM_* was conditionally divided into four parts:

(a) When *φ_CM_* < 0.55, *η_r,_*_0_ progressively increased as *φ_CM_* increased; the dependency *η_r,_*_0_(*φ_CM_*) was described, though only approximately, by the following equation of Krieger and Dougherty
  (8) $$\eta_{r,0}={\left(1+\frac{\varphi_{CM}}{\varphi_{CMmax}}\right)}^{-\left[\eta \right]\varphi_{CMmax}}$$
  where [*η*] is the intrinsic viscosity; the equation was applied with [*η*] = 2.5 (as for hard spheres) and *φ_CMmax_* = 0.55; it should be noted that Equation (8) is essentially different from Equation (6) (which is also commonly applied for hard spheres); however, neither of them can be preferred for describing CM behavior, considering that the fit of experimental data by [6,10] using Equation (6) and by [12] using Equation (8) was rather approximate;
(b) When *φ_CM_* = 0.55–0.61, the increase of $\eta_{r,0}$(*φ_CM_*) slowed down significantly; this was explained by the appearance of direct contacts between confined CMs and the consequent deformation and collapse of their *κ*-casein surface layer. Dahbi et al. [12] concluded that the true voluminosity of CMs decreased, while the true volume fraction of deformed CMs remained nearly constant, as the effective volume fraction of CMs calculated using the assumption *v_CM_* = const. increased from 0.55 to 0.61; it should be noted that this interesting behavior of $\eta_{r,0}$ was not detected by [6];
(c) When *φ_CM_* > 0.61, $\eta_{r,0}$ sharply increased again as *φ_CM_* increased; it was assumed that, although CMs had collapsed brush layers and were in direct contact, shear flow could occur due to the deformation of the CMs under applied stress and possible deswelling and shrinking due to osmotic compression. This behavior was also observed by Bouchoux et al. [6] and agreed with the results of the SAXS experiments [14];
(d) At *φ_CM_* ≈ 0.69, the viscosity diverged because the system jammed; this value of *φ_CM_* agrees with those obtained by [6] (*φ_CM_* ≈ 0.64–0.78) and [10] (*φ_CM_* = 0.71).

Noebel et al. [11] studied the rheological behavior of dispersions prepared by mixing CNI powder (*R_SP_* = 0.10) into synthetic PUF. They obtained shear-flow curves for dispersions with a weight fraction of caseins of 0–0.16 g·g^−1^ and a temperature of 5–35 °C.

In agreement with [6,10], they observed that shear thinning occurred when the CN concentrations exceeded 0.1 g·g^−1^ and that a zero-shear-rate viscosity was reached at the applied shear stress of *τ* ≤ 0.1 Pa for the more concentrated dispersions. The zero-shear-rate relative viscosity was calculated assuming that the viscosity of the dm was constant regardless of the concentration of the dispersed CNI powder and equal to that of a pure solvent (i.e., synthetic PUF).

For all studied temperatures, the obtained dependency $\eta_{rPUF,0}\left(\varphi_{CM}\right)$ (up to *φ_CM_* = 0.6) was well fitted by Equation (8) (with the determination coefficient *r*^2^ = 0.963). The values of the fitting parameters were [*η*] = 4.305 and $\varphi_{CMmax,0}$ = 0.68, regardless of the temperature; thus, it was calculated that *v_CM_* = 4.134 mL·g^−1^ at 20 °C. The value of *φ_CMmax_*_,0_ was consistent with that reported by [6,10,12] for the concentration at the sol–gel transition. However, Noebel et al. [11] did not observe the region of near-constant viscosity reported by [12]. In addition, the obtained value of [*η*] was much higher than that expected for hard spheres (i.e., 2.5). They attributed this difference to the formation of rotating pairs of CMs (dumbbells) that increased the hydrodynamic volume of the moving species when *φ_CM_* = 0.15–0.60. If this hypothesis is accurate, it is unclear why Equation (8), with [*η*] = 4.305, described the influence of concentration on viscosity well for *φ_CM_* < 0.15 (where the dumbbells’ formation must be less probable) and whether the obtained value of voluminosity should be attributed to the dumbbells or to individual CMs. In addition, it was observed that Equation (8) was unable to describe the relative viscosity beyond the zero-shear region.

In 2016, Noebel et al. [15] extended the rheological measurements to a temperature range of 35–70 °C. They used native CM dispersions (*R_SP_* = 0.10) prepared by diluting retentates from skim milk microfiltration with synthetic PUF.

The dependence of *η_rPUF_* on casein concentrations (0.01–0.16 g·g^−1^) (measured at *τ* = 0.1 Pa, at which *η_rPUF_*$\left(\dot{\gamma }\right)$ leveled off for all studied dispersions) was described well by Equation (8) with [*η*] = 3.2 and $\varphi_{CMmax,0}$ = 0.68 (*r*^2^ = 0.99). They attributed the large difference between this value of [*η*] and that in their previous study (3.20 vs. 4.305) to the source of CMs (i.e., skim milk retentate vs. CNI powder, respectively) and the difference between the estimated and theoretical values of [*η*] (3.2 and 2.5, respectively) again to the formation of dumbbells. Interestingly, *η_rPUF_*(*φ_CM_*) was also described well by the equation of Mendoza and Santamaria-Holek [16]:

(9) $$\eta_{r}={\left(1+\frac{\varphi_{CM}}{1-C\varphi_{CM}}\right)}^{-\left[\eta \right]}$$

where

(10) $$C={\varphi_{CMmax}}^{-1}-1$$

with [*η*] = 3.1 and $\varphi_{CMmax,0}$ = 0.68 (*r*^2^ = 0.99). Although both fits had the same determination coefficient *r*^2^ = 0.99, Equation (9) greatly overestimated *η_rPUF_* for *φ_CM_* > 0.4. This emphasizes that *r*^2^ is not a suitable criterion for assessing the goodness of fit of strongly nonlinear dependencies; it also demonstrates the limitations of data fitting in analyzing rheological properties.

Olivarez et al. [17] revised the data of [6,11,12], but only for concentrated dispersions with *φ_CM_* > 0.4, and described shear-flow curves using the equation of Berli and Quemada [18] for concentrated suspensions of microgels:

(11) $$\frac{\eta }{\eta_{\infty }}={\left(\frac{1+\tau /\tau_{c}}{{\left(\eta_{\infty }/\eta_{0}\right)}^{1/2}+\tau /\tau_{c}}\right)}^{2}$$

Unlike Krieger and Dougherty (Equation (5)), Berli and Quemada consider the repulsion between CMs (e.g., due to the *κ*-casein brush), assuming that

(a) The parameter $\tau_{c}$ in Equation (11) is independent of *τ*;
(b) $\tau_{c}$ increases linearly as the repulsion energy between the CMs (*U*) increases:
  (12) $$\tau_{c}=a_{H}^{-3}\left(k_{B}T+U\right)$$
  where *a_H_* is the hydrodynamic radius of CM, and *k_B_T* is the product of the Boltzmann constant and the temperature;
(c) *U* is defined by the distance between the CMs (*h*): e.g., when calculated using the DLVO theory, *U* is practically equal to 0 when *h* exceeds the double thickness of the brush layer (which is ca. 10 nm), and it increases nearly exponentially as *h* decreases below this critical value;
(d) *h* is defined by the volume fraction of CMs.

Therefore, within this approach, for a given CM volume fraction (*φ*_1_), which uniquely defines the distance between CMs (*h*_1_), the viscosity of this dispersion is independent of the function *U*(*h*) for *h* ≠ *h*_1_.

In addition, the model of Berli and Quemada considers a potential decrease in CM volume as the CM concentration increases, which is due to the collapse of the brush layer and osmotic compression of the CM cores under the osmotic stress exerted by the solutes of dm and neighboring CMs.

In agreement with the model of Berli and Quemada, the dependencies $\eta_{\infty }\left(\varphi_{CM}\right)$ and $\eta_{0}\left(\varphi_{CM}\right)$ were described using the equation of Quemada (Equation (6)). However, the values of *φ_CM_* in the original studies [6,11,12], which had been calculated using each study’s constant and different *v_CM_*, were used to analyze the shear-flow curves. Thus, the data analysis did not completely consider potential CM compression at high *φ_CM_*.

The excellent fit of shear-flow curves using Equation (11) enabled $\varphi_{\infty }$, $\varphi_{0}$, and *τ_c_* to be calculated for each dispersion. It must be noted that for a given *φ_CM_*, fitting parameters $\varphi_{\infty }$, $\varphi_{0}$, and *τ_c_* calculated from the shear-flow curves differed greatly among the three studies. Along with the difficulty in determining the voluminosity encountered by [12], this observation challenges the possibility of quantitative comparison of the data on the rheological properties of CM dispersions reported in different studies.

Nonetheless, data from the three studies revealed the following general trends:

(a) $\varphi_{0}$ and $\varphi_{\infty }$ increased as *φ_CM_* increased, which was attributed to a decrease in the hydrodynamic diameter of CMs (i.e., overlapping and collapsing of the *κ*-casein layers and osmotic compression of CMs);
(b) *τ_c_* increased significantly above the certain critical $\varphi_{CM}$, which was attributed to a large increase in interparticle repulsion due to contact and the resulting overlap of brush layers of CMs when the distance between the CM cores decreased to less than 10 nm; it must be noted that this value was obtained assuming that *a_H_* = 71.3 nm and the maximum volume fraction of the cores of CMs = 0.637.

Due to the scattering of estimates of $\varphi_{0}$ and $\varphi_{\infty }$, it is difficult to determine whether $\varphi_{0}$ and $\varphi_{\infty }$ increased significantly before $\varphi_{CM}$ was reached. However, the results of [17] highlight the importance of considering interparticle repulsion when modeling the shear-flow properties of the CM dispersions with $\varphi_{CM}$ > 0.47 (based on data in [11]) or $\varphi_{CM}$ > 0.685 (based on data in [6,12]).

### 1.2. Shear-Flow Properties of Milk Dispersions with Constant High R_SP_

Griffin et al. [19] studied the low-shear relative viscosity of skim milk concentrates produced via ultrafiltration, followed by dilution of the retentate with PUF to achieve the desired CM concentrations (≤0.12 g·g^−1^). They did not report the *R_SP_* of the dispersions, but it was likely similar to the usual *R_SP_* of milk (i.e., ca. 0.2). To calculate the *η_r_* from the apparent viscosity, *η_dm_* was set to *η_PUF_*. Surprisingly, the viscosity of PUF was nearly the same as that of the dm produced via ultracentrifugation of the milk samples.

Equation (6) described the influence of the CN concentration on *η_r_*_,0_ only approximately, but Equation (8), which is also empirical, described it relatively well. Assuming an [*η*] of 2.5 (as for hard spheres), *φ_CMmax_*_,0_ was 0.64, and *v_CM_* was 4.60 mL·g^−1^. Thus, it was concluded that CMs in concentrated milk behave as hard spheres with a constant voluminosity when *φ_CM_* is < 0.55 and thus when the SP concentration in the dm is <0.067 g·g^−1^. Interestingly, the *φ_CMmax_*_,0_ of 0.64 for milk dispersions was close to those mentioned previously for the CM dispersions with a low *R_SP_*: 0.64–0.71 was observed by oscillatory measurements [6,10] or 0.68–0.69, obtained by shear-flow analysis [11,12,15].

In a series of studies, Corredig and co-authors [20,21,22] studied shear-flow properties of milk samples concentrated to different extents via osmotic compression at 4 °C. They assumed that the *v_CM_* remained constant and equal to 4.4 mL·g^−1^ over the studied range of *φ_CM_* = 0.10–0.63. It is worth noting that they prepared samples at temperatures < 12 °C, which can result in the extraction of individual CNs from CMs into the dm. However, they measured concentrations of SPs and total proteins at 25 °C (the temperature of rheological experiments) using standard chemical methods, and the *R_SP_* remained nearly constant (0.21–0.25) (as expected for skim milk) in the region of the CN concentrations in the sample of 0.029–0.139 g·mL^−1^ (calculated using data from [21]).

They observed that the dispersions behaved as Newtonian liquids when *φ_CM_* ≤ 0.4, a threshold that agreed with the results of [6,10] for the CM dispersions in PUF but was significantly higher than that reported in [12] for the CM dispersions in water.

Unlike the observations of Griffin et al. [19], the viscosity of the dm (produced via ultracentrifugation of the samples) increased significantly as the total protein concentration increased. For example, at a total protein concentration of ca. 0.14 g·mL^−1^, the viscosity of a supernatant with an SP concentration of 0.04 g·mL^−1^ was ca. 3–4 times as high as that of PUF (estimated from the data of [21]). The supernatants behaved as Newtonian fluids regardless of the SP concentration.

The dependence of high shear-rate-limiting viscosity, calculated at $\dot{\gamma }$ = 300 s^−1^, at which the viscosity leveled off for all samples studied, on *φ_CM_* was described well (*r*^2^ = 0.999) by Equation (9) with [*η*] = 2.5 and *φ_CMmax,∞_* = 0.81. Despite the high value of *r*^2^, Equation (8) overestimated *η_r,∞_* over the entire range of *φ_CM_*. Therefore, it was concluded that actual *v_CM_* increased as the sample dilution increased. The *φ_CMmax,∞_* of 0.81, larger than that reported in different studies of CM dispersions with a low *R_SP_*, was attributed to the characteristics of the sample itself (concentrated milk); however, the difference with the value reported by [14] was not discussed.

In 2016, Olivares et al. [23] studied the shear-flow behavior of concentrated dispersions of skim milk powder in water (*φ_CM_* = 0.56–0.76 for an assumed constant *v_CM_* = 4.4 mL·g^−1^ and *R_SP_* ≈ 0.22 (according to the literature)). Like in [12], the viscosity of the dm was assumed to equal that of the centrifugal ultrafiltrates of these dispersions, and thus, *η_dm_* may have been underestimated due to the SP rejection during filtration.

In the wide range of shear rates (0.001–1000 s^−1^), shear-flow curves were described well by Equation (11). Low and high shear-rate viscosities were described by Equation (6), and *φ_CM_* was calculated for the assumed constant value of *v_CM_*.

Both $\varphi_{0}$ and $\varphi_{\infty }$ increased significantly as *φ_CM_* increased. This was explained by a decrease in the effective radius of CMs due to hydrodynamic forces exerted on the *κ*-casein brush layers (including the case $\dot{\gamma }$ → 0 since $\varphi_{0}$ increased nearly linearly from 0.59 to 0.75 as *φ_CM_* increased from 0.56 to 0.76). Fitting parameter *τ_c_* increased significantly when $\varphi_{CM}$ exceeded 0.64; this was explained by increasing the repulsions between overlapping *κ*-casein layers (when the distance between the CM cores decreased below 10 nm). For a given distance between CMs (i.e., a given *φ_CM_*), the calculated repulsion energy in the concentrated milk samples (*R_SP_* ≈ 0.22) was one order of magnitude higher than that calculated by [17] based on data from [12] for the CM dispersions in water (*R_SP_* = 0.08) and on data from [6] for the CM dispersions in PUF (*R_SP_* = 0.05). However, it was similar to (if not lower than) that of the CM dispersions in synthetic PUF calculated by [17] based on data from [11] (*R_SP_* ≈ 0.10). Therefore, the role of SP in the short-distance repulsion between CMs was not fully elucidated.

### 1.3. Shear-Flow Properties of Mixed Milk Protein Dispersions with Variable R_SP_

Sauer et al. [24] compared the shear-flow properties of two mixed milk protein dispersions (*R_SP_* = 0.04 and 0.10) prepared by mixing CNI powders in water. The CN concentration ranged from 0.025 to 0.125 (g·g^−1^). For a given CN concentration, dispersions with a higher *R_SP_* had a lower apparent viscosity (data for $\dot{\gamma }$ = 100 s^−1^). This observation is rather unexpected because the dm of the dispersions with *R_SP_* = 0.10 had significantly higher lactose and SP concentrations than that with *R_SP_* = 0.04 and thus must have had a significantly higher apparent viscosity at given CN concentrations if the CMs behave as hard spheres. We attribute this observation to the differing characteristics of the powders used to prepare the dispersions with *R_SP_* values of 0.04 and 0.10.

Few studies have examined mixed milk protein dispersions with different *R_SPs_*, but they have the same source of CMs and SPs. For example, Coskun et al. [25] compared the viscosities of mixed milk protein dispersions produced by micro- and ultrafiltration of skim milk (*R_SP_* = 0.28 and 0.31, respectively). They observed that for a CN concentration of ca. 0.05 g·g^−1^, increasing the *R_SP_* from 0.28 to 0.31 increased the apparent viscosity measured at the arbitrarily chosen $\dot{\gamma }$ = 200 s^−1^ by ca. 30%.

Warncke et al. [26] studied the rheological properties of skim milk retentates that had different SP concentrations. The samples with *R_SP_* = 0.02, 0.08, and 0.15 were produced via micro-diafiltration using different quantities of PUF. Unlike Sauer et al. [24], Warncke et al. [26] observed that the apparent viscosity of dispersions measured at $\dot{\gamma }$ = 1000 s^−1^ increased as the *R_SP_* decreased for a given total protein concentration in the dispersion (0.03–0.13 g·g^−1^). Unfortunately, they described the shear-flow curves using only the power law equation (Ostwald–de Waele):

(13) $$\eta =K{\dot{\gamma }}^{n}$$

with empirical coefficients *K* and *n*.

In summary:

(a) The most recently published data on the shear-flow behavior of mixed milk protein dispersions were analyzed using different, usually empirical equations; thus, their results are difficult to compare;
(b) Various simplifications were made concerning the viscosity of the dm and constancy of CM voluminosity;
(c) The results seemed to be influenced strongly by the source of CMs and often varied within a given study;
(d) The most reliable results (that CMs behave as hard spheres with constant voluminosity at a low *φ_CM_* [6] and repulsion between CMs increases as the *R_SP_* increases at high CM concentrations [17]) were not verified for different SP concentrations;
(e) In addition, the influence of the possible absorption of SPs by CMs on rheological properties of mixed dispersions and their dm was not discussed.

Therefore, the main objective of the present study was to describe and compare the rheological properties of mixed dispersions of CMs and SPs that had different relative SP concentrations (i.e., *R_SP_*). Dispersions with different *R_SP_* were prepared by mixing concentrated dispersions of CNI and WP isolate powders in PUF at different weight ratios. The CM concentration of the dispersions varied due to the PUF diluting the mixed dispersions or to osmotic compression. Obtained dispersions were characterized through absorption and rheological (shear-flow and oscillatory) experiments.

We focused on the following research questions:

(a) Are SPs absorbed by CMs, and does the possible distribution of SPs between CMs and the dm influence the viscosity of the dm?
(b) How to describe the dependence of dm viscosity on the SP concentration?
(c) How to describe the viscosity of mixed dispersions at different *R_SP_* and different CM concentrations, and how does the *R_SP_* influence the viscosity of mixed dispersions at different shears?
(d) Does the presence of SPs influence the sol–gel transition concentration in mixed dispersions?

The results would be useful for modeling and optimizing industrial operations of the filtration–separation of skim milk and concentration–evaporation of milk protein dispersions.

## 2. Materials and Methods

### 2.1. Raw Materials

CNI powder “Casein isolate 88%,” and WP isolate powder “Pronativ” (WPI) were provided by Lactalis, France. The manufacturing of the CNI and WPI involves the microfiltration of skim milk, which ensures that all CMs remain in the retentate, followed by dia-microfiltration of the retentate with water (aimed at the additional separation of CMs from SPs and other solutes), concentration–evaporation, and spray drying; WPI is produced by ultrafiltration of the permeate from microfiltration (aimed at concentrating SPs and removing low-molecular-weight compounds), followed by spray drying.

The PUF was produced at the STLO Dairy Platform (Rennes, France) by ultrafiltration of fresh skim milk with a spiral-wound polymeric membrane module (UP010XT, molecular weight cut-off of 10 kDa; Microdyn-Nadir, Wiesbaden, Germany) at 8 °C. After preparation, the PUF was stabilized by adding 0.02 wt.% of NaN_3_ (Sigma-Aldrich, Saint-Louis, MO, USA) and then stored at 4 °C.

### 2.2. Composition of Raw Materials

The CNI, WPI, and PUF were analyzed as follows: the dry matter (DM) content was determined using the weight method after overnight drying at 104 °C, and the dried samples were used to determine the ash (total mineral (MN)) content via incineration at 550 °C for 5 h [27].

The quantities of total-nitrogen- (TN), non-casein-nitrogen- (NCN), and non-protein-nitrogen-containing compounds (NPN, attributed to compounds with a molecular weight < 10 kDa: peptides, urea, vitamins) in the CNI and WPI were determined using the conventional method that involves the isoelectric precipitation of CNs and WPs followed by Kjeldahl analysis [28,29]. Before the analysis, the CNI and WPI powders were dispersed in distilled water at a concentration of about 0.025 g∙g^−1^: the CNI dispersion was vigorously stirred at 36 °C for 15 h, while the WPI was dissolved by gently stirring it at room temperature. The TN, NCN, and NPN contents were determined using conversion factors of 6.38, 6.25, and 6.19, respectively [28,29]. The CN content was calculated as the TN content minus the NCN content, while the WP content was calculated as the NCN content minus the NPN content.

The compositions of the CNI, WPI, and PUF are presented in Table 1.

It must be noted that:

(a) WPs detected in the CNI (5.46 wt.%) were those that remained in the retentate of skim milk after dia-microfiltration;
(b) The quantity of sCNs that remain in the CNI prepared by the standard method of “hot” microfiltration is usually negligible compared to those of CNs (*ω_CN_*) and WPs (*ω_WP_*); therefore, all detected caseins were assumed to be micellar (*ω_mCN_*), and the quantity of SPs (WPs + sCNs (*ω_SP_*)) was equated to *ω_WP_*;
(c) Usually, the WPI produced using standard “hot” microfiltration contains almost no mCNs; this was confirmed by the low turbidity of the WPI dispersions; thus, the CNs detected in the WPI (7.92 wt.%) were assumed to be sCNs that entered the permeate during micro- and dia-microfiltration of skim milk; therefore, the quantity of SPs (WPs + sCNs) in the WPI was calculated as the sum of *ω_CN_* and *ω_WP_* estimated via Kjeldahl analysis (7.92 + 79.53 = 87.45 (wt.%)), and the number of mCNs (*ω_mCN_*) in the WPI was set to 0.

The total protein content (*ω_PR_*) was defined as *ω_mCN_* + *ω_SP_*. The water (w) content ($\omega_{w}$) was calculated from the total DM content: $\omega_{w}$ = 1 − $\omega_{DM}$. The quantity of low-molecular-weight organic compounds (including lactose) (*ω_o_*) was determined as *ω_o_* = *ω_DM_* − (*ω_PR_* + *ω_MN_*).

### 2.3. Preparation of Mother Dispersions of Milk Proteins

A concentrated (mother) dispersion of the CNI was prepared by thoroughly stirring the CNI powder into the PUF at 36 °C for 15 h. According to [30], this results in the complete solubilization of the CNI powder, disruption of CM aggregates, and swelling of CMs to their original size. A concentrated (mother) dispersion of the WPI was prepared by gently dissolving the WPI powder in the PUF at room temperature for 3–4 h. Before dispersing the powders, the PUF was additionally stabilized by adding NaN_3_ and thiomersal up to their final concentrations of 0.05 wt.% and 0.02 wt.%, respectively.

The mother dispersions of the CNI and WPI were mixed at different weight ratios and gently shaken overnight at 20 °C to obtain concentrated mixed (mother) dispersions of milk proteins with different *R_SP_*.

In total, four concentrated milk protein dispersions with different *R_SP_* were prepared: *R_SP_* = 0.066 (CNI mother dispersion), *R_SP_* = 0.158 and 0.214 (mother mixed protein dispersions), and *R_SP_* = 1 (WPI mother dispersion). The total protein content of the mother dispersions ($\omega_{PR}$) ranged from 0.125 (CNI) to 0.174 (WPI).

### 2.4. Preparation of Diluted Milk Protein Dispersions

Liquid mixed milk protein dispersions with different *R_SP_* and a $\omega_{PR}$ < 0.125 were prepared by gently mixing each mother dispersion with the PUF (overnight at 20 °C). Since the PUF did not contain proteins, diluting the mixed protein dispersions did not change the *R_SP_*.

The pH value of all prepared dispersions (including mother dispersions) was in the range of 6.7 ± 0.1 at 20 °C (i.e., close to that of fresh skim milk).

### 2.5. Separation of the Dispersion Medium

CMs were separated from the dm via ultracentrifugation at the centrifugal rotation speed of 3·10^4^ rpm (centrifugal acceleration ≈ 8.1·10^4^× *g*) at 20 °C for 3 h. Samples of ca. 16 mL were centrifuged in 32 mL Beckman Coulter tubes (rotor model 50.2Ti, centrifuge model Sorvall Discovery 90SE, Waltham, MA, USA). After the sedimentation of CMs, clear supernatants were sampled using a pipette and used to determine the viscosity and total DM content.

### 2.6. Concentrating Milk Protein Dispersions via Osmotic Compression

Protein dispersions with $\omega_{PR}$ > 0.125 were prepared via osmotic compression of the mixed milk protein dispersions described above. The method of osmotic compression is based on the exchange of the aqueous phase (water, lactose, minerals) between the protein dispersion in a dialysis bag and a reservoir filled with a stressing polymer solution of known osmotic pressure; the method is explained in detail elsewhere [6,10]. In brief, Spectrapor dialysis bags, made of regenerated cellulose membrane with a molecular weight cut-off of 6–8 kDa, having a diameter of 25.5 mm (Repligen, Waltham, MA, USA), were filled with ca. 10 mL of liquid suspension with a given *R_SP_*. Closed bags were placed in ca. 500 mL of the stressing polymer solution prepared by dissolving polyethylene glycol (PEG) (20 kDa, Sigma-Aldrich, Saint-Louis, MO, USA) in PUF (with 0.05 wt.% of NaN_3_ and 0.02 wt.% of thiomersal added). The molecular weight cut-off of the dialysis bag material and the PEG molecular weight were both chosen to ensure that all proteins were retained in the bag and that low-molecular-weight compounds (e.g., water, lactose, minerals, small peptides) passed freely into the stressing solution while preventing PEG from diffusing into the compressed dispersions. Therefore, the *R_SP_* did not change during osmotic compression because the dialysis bag material was impermeable to SPs and CNs.

The osmotic pressure of the PEG solutions (*π*) was calculated using Cohen’s equation [31]

(14) $$\pi =\frac{R_{g}T}{M_{m}\bar{V}}{\left(\frac{M_{m}}{M}\right)}^{5/9}\left[\frac{C}{C_{N}}+\alpha {\left(\frac{C}{C_{N}}\right)}^{9/4}\right]$$

where *R_g_* is the universal gas constant, *T* is the temperature, *M_m_* and *M* are the molecular weights of the PEG monomer and polymer, respectively, $\bar{V}$ is the partial specific volume of PEG, C^*^ is the PEG concentration, *α* is the crossover index, and $C_{N}$ is the characteristic polymer concentration, which depends on the number of monomers per polymer chain. According to [31], *α* = 0.434 and $C_{N}$ = 9.07 kg·m^−3^ for the PEG used.

Mother dispersions (CNI, WPI, and mixed dispersions) were compressed at osmotic pressures of 50, 100, and 200 (kPa), while diluted dispersions with $\omega_{PR}$ ≈ 0.065 were compressed at 12.5 and 25 (kPa).

Dialysis bags with samples were equilibrated with PEG solutions at 20 °C under gentle shaking at 90 rpm. During two weeks of compression, small quantities of each liquid dispersion were added to the bags to produce final samples large enough for rheological measurements. Then, the bags were replaced in the freshly prepared PEG solutions with the same *π* and left for one week without refilling. The pH of the PEG solutions, measured three times per week, did not decrease over the three weeks of the osmotic compression experiment (pH = 6.7 ± 0.1).

The DM content of the compressed dispersions was determined using the weight method after overnight drying at 105 °C.

### 2.7. Shear Flow and Oscillatory Rheology

All rheological measurements were performed at 20 °C.

The rheological properties of the liquid dispersions and supernatants were characterized by measuring steady-state shear-flow curves. A viscometer (Low Shear 400, Lamy Rheology, Champagne au Mont d’Or, France) with Couette geometry (inner and outer radius of 5.5 and 6.0 (mm), respectively) was used to characterize the samples with an apparent viscosity < 10 mPa·s at shear rates of 1–120 s^−1^. More concentrated and viscous liquid samples (that did not reach the high shear-rate-limiting viscosity at $\dot{\gamma }$ = 100 s^−1^) were characterized using a rheometer (DHR2, TA Instruments, Guyancourt, France) with a stainless-steel cone–plate geometry (cell diameter = 50 mm, cone angle = 2°) at shear rates of 1–1000 s^−1^. A cover and mineral oil were used to prevent evaporation during the measurements with the rheometer.

After the apparent viscosity at a constant shear rate reached a constant value (usually, after 1–5 min of shear application), the shear rate was increased incrementally. After the highest shear rate was reached, measurements at shear rates of 3 and 30 (s^−1^) were repeated. For all the samples studied, no hysteresis was observed at these shear rates.

The viscosity of the dispersions relative to PUF viscosity (*η_rPUF_*) was calculated as

*η_rPUF_* = *η*/*η_PUF_* (15)

with *η_PUF_* = 1.26 ± 0.04 (mPa·s), according to the viscometer at 20 °C.

The same rheometer equipped with a stainless-steel plate–plate cell with a grooved surface (diameter = 20 mm) was used to measure the viscoelastic properties of the concentrated samples. Elastic (*G*′) and loss (*G*″) moduli were first determined as functions of stress at a frequency of 1 Hz. Then, a strain of 1%, located in the linear viscoelastic zone, was applied at frequencies (*θ*) of 0.1–10 Hz.

The shear-flow or oscillatory properties were measured twice for each obtained sample. Means and standard deviations were calculated; the latter are presented in the figures as error bars.

### 2.8. Data Analysis

Least-square fitting of the experimental data and a calculation of 95% prediction intervals were performed using the Table Curve 2D software v. 5.01 (SYSTAT Software Inc., Chicago, IL, USA).

### 2.9. Composition of Dispersions and Determination of Component Distribution Between CMs and dm

Appendix A describes the methods applied to determine (1) the distribution of the soluble components (SPs, eMN, o) and w between the swelled CMs and dm in all the dispersions studied and (2) the total composition of the dispersions prepared via osmotic compression.

## 3. Results

### 3.1. Distribution of Solutes in Dispersions and Composition of the Dispersion Medium

The impact of the possible absorption of the solutes (SPs, o, and eMN) by CMs on the concentrations of these solutes in the dm must increase as the CM concentration in the dispersions increases. In addition, the impact of the possible absorption of SPs on the total DM content of the dm must increase as the *R_SP_* increases. Therefore, to determine whether different solutes were absorbed by CMs in the studied dispersions, the dm of the mother dispersions (as well as several less concentrated dispersions) with different *R_SP_* was separated from the CMs by ultracentrifugation, and the total DM content of the dm (the supernatant) ($\omega_{DM,exp}^{dm}$) was measured.

The theoretical total DM content of the dm ($\omega_{DM,th}^{dm}$) was calculated using Equation (A10) (Appendix A), assuming $v_{CM}$ of 4.10 mL·g^−1^. The calculations were performed using different assumptions about the ability of CMs to absorb SPs, o, and/or eMN. This ability was characterized via the parameters *f_SP,_ f_o_*, and *f_eMN_*, respectively, introduced and discussed in Appendix A, Equation (A2). In brief, *f_i_* is the concentration of solute *i* in water absorbed by the CMs divided by that in the water of the dm. Table 2 presents eight sets of assumptions for which the $\omega_{DM,th}^{dm}$ was calculated: each set, designated by a letter from A to H, corresponds to a certain combination of assumed values of *f_i_*, where *i* stands for SP, o, and eMN, and the values of *f_i_* are either equal to 0 or 1: *f_i_* = 0 implies that component *i* is not absorbed by CM, while *f_i_* = 1 implies an equal ability of water absorbed by CMs and water of the dm to dissolve component *i*.

**Figure 1:**
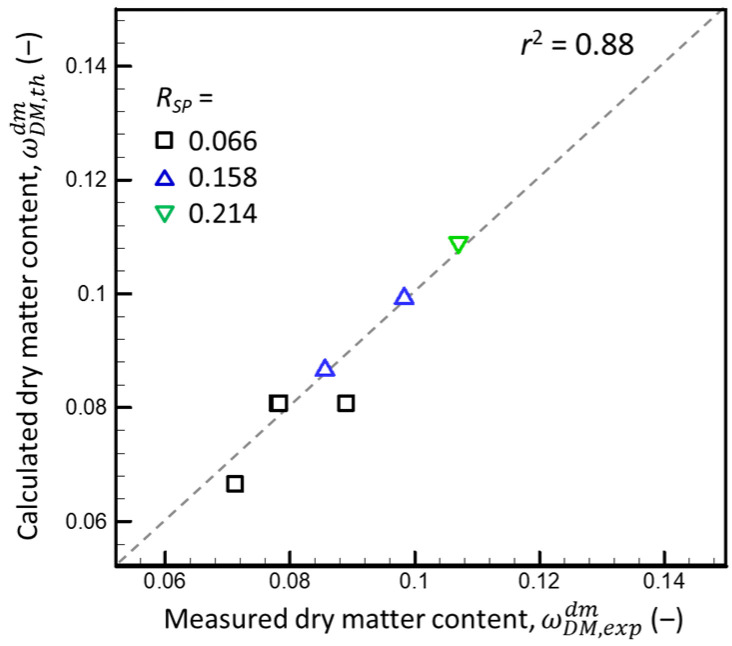
Theoretical DM content of the dispersion medium ($\omega_{DM,th}^{dm}$) (calculated under the assumption set C (Table 2) vs. measured total DM content of supernatants ($\omega_{DM,exp}^{dm}$) of mixed milk protein dispersions). Data for suspensions with *R_SP_* = 0.066 and $\omega_{PR}^{D}$ = 0.083 and 0.125 (squares), *R_SP_* = 0.158 and $\omega_{PR}^{D}$ = 0.105 and 0.126 (triangles), and *R_SP_* = 0.214 and $\omega_{PR}^{D}$ = 0.126 (inverted triangles). The dashed line corresponds to $\omega_{DM,exp}^{dm}$ = $\omega_{DM,th}^{dm}$. The determination coefficient for the data fitting by this equation (*r*^2^) is shown near the dashed line. The DM content at the origin is that of PUF (0.0521).

The total DM content of the supernatants produced via ultracentrifugation ($\omega_{DM,exp}^{dm}$) vs. the theoretical DM content of the dm of corresponding dispersions ($\omega_{DM,th}^{dm}$) varied among the eight sets of assumptions (Figure S1, Supplementary Materials). The results for assumption set C (i.e., *f_SP_* = 0, *f_o_* = 1, *f_eMN_* = 0) had the highest *r*^2^ between experimental and theoretical $\omega_{DM}^{dm}$ (i.e., 0.88) (Figure 1).

This result suggests that the CMs absorbed only low-molecular-weight organic compounds (represented mainly by lactose) (i.e., the absorbed water and that of the dm had the same relative quantity of o, *f_o_* = 1). It also suggests that *f_SP_* = 0 (i.e., all SPs in the dispersions were in the dm).

Note that analyzing the DM content of the dm cannot rule out other potential situations when CMs absorb different solutes of the dm simultaneously with *f_i_* < 1. For example, moderate absorption of SPs by CMs (*f_SP_* < 1) can compensate for a lower absorption of o (*f_o_* < 1) and result in the same decrease in the DM content of the dm as that predicted with assumption set C (*f_SP_* = 0 and *f_o_* = 1). Thus, another property of dm—dynamic viscosity—was studied to confirm assumption set C.

### 3.2. Shear-Flow Properties of WPI Dispersions and Supernatants

**Figure 2:**
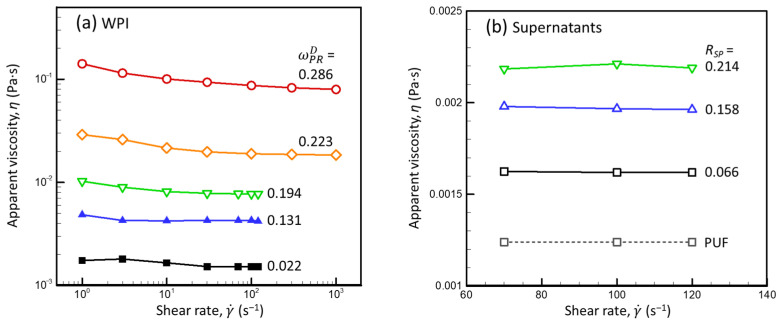
(**a**) Steady-state apparent viscosity (*η*) as a function of shear rate ($\dot{\gamma }$) for WPI dispersions (*R_SP_* = 1) prepared by mixing WPI powder with PUF (solid symbols) and following osmotic compression (open symbols); total protein concentrations ($\omega_{PR}^{D}$) are shown next to the curves. (**b**) η(γ|) for supernatants produced via ultracentrifugation of mother dispersions; the *R_SP_* in dispersions used for ultracentrifugation are shown next to the curves; the dashed line indicates experimental data for PUF.

Figure 2 presents examples of the steady shear-flow curves for the WPI dispersions with various $\omega_{PR}^{D}$ (calculated via Equations (A12) and (A27), Appendix A) (Figure 2a), and those for supernatants (obtained from the mother dispersions of milk proteins with *R_SP_* = 0.066–0.214 and $\omega_{PR}^{D}$ = 0.125–0.126) (Figure 2b).

The apparent viscosity of all the WPI dispersions decreased as the shear rate increased (Figure 2a). A high shear-rate-limiting viscosity was reached at $\dot{\gamma }$ = 70 s^−1^ for the dispersion with $\omega_{PR}^{D}$ < 0.223 and at $\dot{\gamma }$ = 300 s^−1^ for the dispersion with $\omega_{PR}^{D}$ = 0.223, while the dispersion with $\omega_{PR}^{D}$ = 0.286 did level off, even at 1000 s^−1^. Apparent viscosities of the PUF and of the supernatants obtained from the mother dispersions were nearly constant at $\dot{\gamma }$ = 70–120 s^−1^, regardless of the *R_SP_* (Figure 2b).

**Figure 3:**
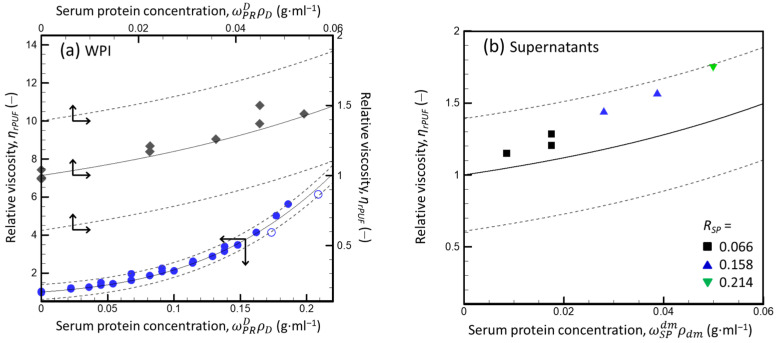
(**a**) Relative viscosity ($\eta_{rPUF}$) at a shear rate of 100 s^−1^ as a function of SP concentration ($\omega_{PR}^{D}\rho_{D}$) in liquid (solid symbols) and compressed (open symbols) dispersions of WPI; solid curve corresponds to data fitting by Lee’s equation, dashed curves correspond to lower and upper limits of the 95% prediction interval; left and bottom axes correspond to results over the entire range of $\omega_{PR}^{D}\rho_{D}$ studied (circles), while the right and top axes correspond to results for the lowest $\omega_{PR}^{D}\rho_{D}$ (diamonds). (**b**) Relative viscosity ($\eta_{rPUF}$) at a shear rate of 100 s^−1^ as a function of SP concentration ($\omega_{SP}^{dm}\rho_{D}$) in supernatants produced via ultracentrifugation of dispersions with different *R_SP_* and $\omega_{PR}^{D}$: *R_SP_* = 0.066 and $\omega_{PR}^{D}$ = 0.083 and 0.125 (squares), *R_SP_* = 0.158 and $\omega_{PR}^{D}$ = 0.105 and 0.126 (triangles), and *R_SP_* = 0.214 and $\omega_{PR}^{D}$ = 0.126 (inverted triangles); solid and dashed curves are the same as in (**a**).

As expected, the apparent viscosity of the WPI dispersions increased as the $\omega_{PR}^{D}$ increased. To quantify this behavior, relative viscosities of dispersions with $\omega_{PR}^{D}$ < 0.223 were calculated from *η* measured at 100 s^−1^ using Equation (15). The dependence of $\eta_{rPUF}$ on the concentration of SPs (including sCNs) was described using Lee’s equation [32]:

(16) $$\eta_{r}=1+2.5\varphi +7.031\varphi^{2}+37.371\varphi^{3}$$

where $\eta_{r}$ is the viscosity of the dispersion relative to that of the dm, and φ is the particle volume fraction. For the WPI dispersions in PUF, Equation (16) can be rewritten as

(17) $$\eta_{rPUF}=1+2.5\left(v_{SP}\omega_{PR}^{D}\rho_{D}\right)+7.031{\left(v_{SP}\omega_{PR}^{D}\rho_{D}\right)}^{2}+37.371{\left(v_{SP}\omega_{PR}^{D}\rho_{D}\right)}^{3}$$

where $\rho_{D}$ is the density of the WPI dispersion (depending on $\omega_{PR}^{D}$, calculated using Equation (A31), Appendix A), and $v_{SP}$ is the SP voluminosity (fitting parameter, assumed to be independent of $\omega_{PR}^{D}$). Because the product $\omega_{PR}^{D}\rho_{D}$ equals the protein concentration in the dispersion (measured in g·mL^−1^), the product $v_{SP}\omega_{PR}^{D}\rho_{D}$ equals the effective volume fraction of proteins (here, SPs). The result of the data fitting is shown in Figure 3a.

Since the open symbols in Figure 3a are within the prediction interval of the dependence obtained for the solid symbols, it can be concluded that the viscosity of the WPI dispersions was not impacted by the preparation method. For the wide range of SP concentrations in liquid and compressed WPI dispersions ($\omega_{PR}^{D}\rho_{D}$ ≤ 0.21), the influence of $\omega_{PR}^{D}\rho_{D}$ on $\eta_{rPUF}$ was fitted fairly well by Equation (17) (*r*^2^ = 0.98). In agreement with the meaning of Lee’s equation, this implies that, within the given concentration range, the rheological behavior of the WPI dispersions can be approximated as that of a system of hard spheres with only hydrodynamic interactions with the dm (here, PUF), individual particles (SPs), and temporary particle pairs and triplets [32,33]. The obtained value of the fitting parameter $v_{SP}$ (2.09 ± 0.02 (mL·g^−1^)) was close to that reported for globular proteins [33].

Figure 3b presents the relative viscosities of supernatants (calculated for $\dot{\gamma }=100s^{-1}$ using Equation (15)) as a function of the SP concentration in the dm ($\omega_{SP}^{dm}\rho_{dm}$, where $\omega_{SP}^{dm}$ is the weight fraction of the SP in the dm calculated using Equation (A9) and the set of assumptions *f_SP_* = 0, *f_o_* = 1, *f_eMN_* = 0, and *v_CM_* = 4.10 mL·g^−1^, and $\rho_{dm}$ is the density of the dm calculated by applying Equation (A31) to the known composition of the dm (Appendix A)). For different dispersion compositions (*R_SP_* and $\omega_{PR}^{D}$), the estimated $\eta_{rPUF}\left(\omega_{SP}^{dm}\rho_{dm}\right)$ (Figure 3b) were close to $\eta_{rPUF}\left(\omega_{PR}^{D}\rho_{D}\right)$ for the WPI dispersions (Figure 3a, solid diamonds). The measured $\eta_{rPUF}$ for the supernatants were systematically higher than those predicted by Equation (17), with $v_{SP}$ = 2.09 ± 0.02 (mL·g^−1^), probably due to the presence of residual “mini” CMs after 3 h of ultracentrifugation: it is likely that the centrifugal separation of small CMs became less effective as the viscosity of the serum increased as the *R_SP_* increased. Nonetheless, the $\eta_{rPUF}\left(\omega_{SP}^{dm}\rho_{dm}\right)$ calculated for all supernatants lay within the 95% prediction interval based on the WPI data described by Lee’s equation (Figure 3b).

The data presented in Figure 3b also confirm the validity of assumption set C and suggest that the total SPs influenced the rheological properties of the dm of mixed dispersions in the same way that they influenced the viscosity of the WPI dispersions in the absence of CMs (i.e., mainly via hydrodynamic interactions between individual proteins). This rules out the assumption [*f_SP_* > 0, *f_o_* < 1] discussed above because the hypothetical increase of *f_SP_* above 0 would be equivalent to a decrease in the SP concentration in the dm, and thus, a decrease in $\omega_{SP}^{dm}\rho_{D}$ that would shift $\eta_{rPUF}\left(\omega_{SP}^{dm}\rho_{dm}\right)$ to the left (i.e., outside the upper prediction interval) (Figure 3b). Thus, *f_SP_* = 0, *f_o_* = 1, and *f_eMN_* = 0 were used in the following calculations, and Equation (17) with $v_{SP}$ = 2.09 ± 0.02 (mL·g^−1^) was used to calculate the serum viscosity for dispersions with $\omega_{SP}^{dm}$ ≤ 0.05 (i.e., up to the upper limit of $\omega_{SP}^{dm}$ in Figure 3b).

It must be noted that our attempt to describe the shear-flow behavior of SPs over the entire range of $\varphi_{SP}^{D}$ and $\dot{\gamma }$ with the help of the commonly used Equations (6) and (9) is presented in Supplementary Materials (Figures S2 and S3). For the $\dot{\gamma }$ studied, the influence of $\varphi_{SP}^{D}$ on $\eta_{rPUF}^{WPI}$ was not described adequately by Equations (6) and (9) with a single value of the critical SP volume fraction (at which the system’s viscosity diverges (*φ_m_*) [34]) in the entire range of $\varphi_{SP}^{D}$.

### 3.3. Steady-State Shear-Flow Properties of CNI and Mixed Milk Protein Dispersions, Region of Newtonian Behavior

**Figure 4:**
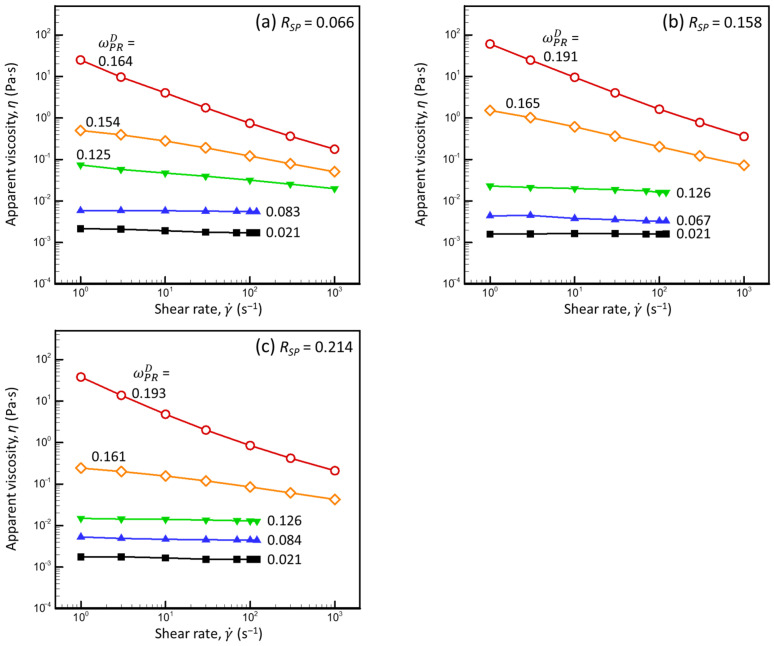
Steady-state apparent viscosity (*η*) as a function of shear rate ($\dot{\gamma }$) for dispersions with *R_SP_* = 0.066 (**a**), 0.158 (**b**), and 0.214 (**c**): solid symbols—liquid dispersions prepared by mixing and diluting CNI and WPI powders with PUF; open symbols—compressed dispersions prepared via osmotic compression; values of $\omega_{PR}^{D}$ are shown next to the curves.

Figure 4 presents the shear-flow curves of the CNI dispersions (*R_SP_* = 0.066) and mixed milk protein dispersions (*R_SP_* = 0.158 and 0.214) with different total protein concentrations ($\omega_{PR}^{D}$) (calculated using Equation (A12) for the liquid dispersions and Equations (2), (3), and (A24) for the compressed dispersions, assuming *v_CM_* = 4.10 mL·g^−1^).

The viscosity of the mixed milk protein dispersions expectedly increased as the total protein concentration increased and decreased as the shear rate increased (Figure 4). The less concentrated dispersions nearly reached the high shear-rate-limiting viscosity. The maximum $\omega_{PR}^{D}$, at which the high shear-rate-limiting viscosity was reached at $\dot{\gamma }$ = 100 s^−1^, increased as the *R_SP_* increased; thus, the increase in SP concentration yielded dispersions with “more Newtonian” behavior for a given $\omega_{PR}^{D}$. Based on these results, this phenomenon was independent of the method used to prepare the dispersion (powder dispersion or osmotic compression).

The rheological behavior of the dispersions with near-Newtonian behavior at high shear rates was analyzed using a modified Lee’s equation (Equation (18)). Since the dispersions contained SPs that increased the viscosity the of dm, the relative viscosity of the dispersions was described as

(18) $$\eta_{rdm}^{D}=\frac{\eta_{rPUF}^{D}}{\eta_{rPUF}^{dm}}=\frac{1+2.5\varphi_{CM}^{D}+7.031{\left(\varphi_{CM}^{D}\right)}^{2}+37.371{\left(\varphi_{CM}^{D}\right)}^{3}}{1+2.5\varphi_{SP}^{dm}+7.031{\left(\varphi_{SP}^{dm}\right)}^{2}+37.371{\left(\varphi_{SP}^{dm}\right)}^{3}}$$

where $\eta_{rdm}^{D}$ is the viscosity of the dispersion relative to the viscosity of the dm, and $\eta_{rPUF}^{D}$ and $\eta_{rPUF}^{dm}$ are the viscosities of the dispersion and its dm relative to the PUF viscosity, respectively. The CM volume fraction in the dispersion ($\varphi_{CM}^{D}$) and the SP volume fraction in the dispersion were calculated using Equations (2), (3) and (A30) for *v_SP_* = 2.09 mL·g^−1^ (Figure 3) and different assumed values of *v_CM_* in the vicinity of 4.10 mL·g^−1^.

**Figure 5:**
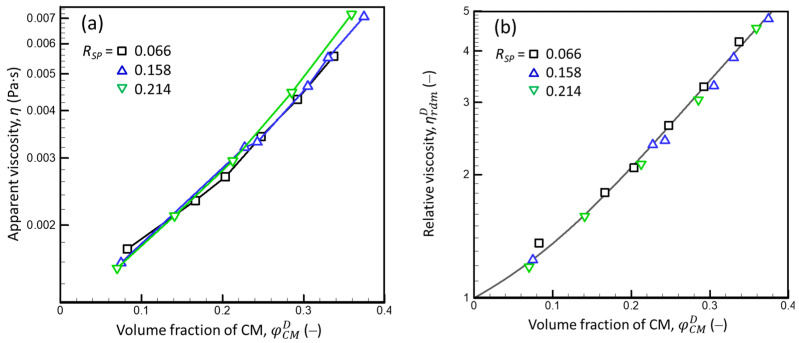
Apparent (*η*) (**a**) and relative ($\eta_{rdm}^{D}$) (**b**) viscosity of dispersions with near-Newtonian behavior at $\dot{\gamma }=100s^{-1}$ as a function of $\varphi_{CM}^{D}$ calculated for *v_CM_* = 4.15 mL·g^−1^: *R_SP_* = 0.066 (squares), 0.158 (triangles), and 0.214 (inverted triangles); the solid curve in Figure 5b was calculated using the modified Lee’s equation (Equation (18)).

Figure 5 presents the dependencies of apparent viscosity (η) (a) and relative viscosity ($\eta_{rdm}^{D}$) (b) on $\varphi_{CM}^{D}$ for dispersions with an *R_SP_* = 0.006, 0.158, and 0.214; $\varphi_{CM}^{D}$ was calculated using *v_CM_* = 4.15 ± 0.04 (mL·g^−1^), which resulted in Equation (18) describing the data best (*r*^2^ > 0.99 for each *R_SP_*). For a given $\varphi_{CM}^{D}$, the difference between the apparent viscosities of dispersions with different *R_SPs_* increased as $\varphi_{CM}^{D}$ increased: *η*(*R_SP_* = 0.066) < *η*(*R_SP_* = 0.021) (Figure 5a). This can be due to an increase in the difference in the viscosity of dm, which increased when the difference in $\varphi_{SP}^{dm}$ increased as $\varphi_{CM}^{D}$ increased at a given *R_PS_*. However, the relative viscosities of the dispersions ($\eta_{rdm}^{D}$) were nearly independent of $\varphi_{CM}^{D}$ and were described by Equation (18) with a single value of the fitting parameter *v_CM_*. The fairly good correspondence between the obtained (symbols) and theoretical (solid curve) dependences implies that, despite the nature of CMs, their rheological behavior at $\dot{\gamma }=100s^{-1}$ can be described as that of hard spheres, showing only the hydrodynamic interactions CM–CM and CM–dm. This description was valid for $\omega_{SP}^{dm}$ ≤ 0.036 ($\varphi_{SP}^{dm}$ ≤ 0.077), which was the SP concentration in the dm of the most concentrated dispersion with *R_SP_* = 0.214 that still demonstrated near-Newtonian behavior at $\dot{\gamma }=100s^{-1}$.

Interestingly, the value *v_CM_* = 4.15 mL·g^−1^ was practically equal to that reported in [11] for the reconstituted dispersions of CMs (i.e., 4.13 mL·g^−1^). However, as explained above, the latter was calculated using a different approach: fitting the dependence of a low-shear-limiting viscosity on $\varphi_{CM}^{D}$ (for $\varphi_{CM}^{D}$ = 0–0.6) using the equation of Krieger and Dougherty (Equation (8)). This approach did not consider the potential influence of SPs on the viscosity of the dm and relied on the use of the fitting constants 4.3 (for the intrinsic viscosity) and 0.68 (for the maximum particle-packing volume fraction). Lee’s equation, which is applied in our study, does not rely on the maximum particle volume fraction and uses the theoretical value of intrinsic viscosity for the dispersion of hard spheres (i.e., [*η*] = 2.5) (Equation (18)). Thus, the agreement between the *v_CM_* calculated in the present study and those reported in [11] should be considered accidental.

The best fit for CM voluminosity (*v_CM_*) of 4.15 mL·g^−1^ was used in the following data analysis. It must be noted that applying the *v_CM_* of 4.15 mL·g^−1^ instead of 4.10 mL·g^−1^ when calculating the DM content and SP concentration in the dm of the CNI and mixed protein dispersions practically did not impact the conclusion about the component distribution in the dispersions. Moreover, the rheological properties of the supernatants (i.e., the results presented in Figure 1, Figure 3b, Figure 5a and Figure S1 (Supplementary Materials)) were practically not impacted by changing the *v_CM_* from 4.10 to 4.15 (mL·g^−1^).

### 3.4. Steady-State Shear-Flow Properties of CNI and Mixed Milk Protein Dispersions, Entire Range of CM Concentrations and Shear Rates

For most of the dispersions studied, the influence of $\dot{\gamma }$ on *τ* was described well (*r*^2^ ≥ 0.99) by the commonly used empirical Equation (13) (see the Supplementary Materials for the values of the fitting parameters *K* and *n* obtained). However, we did not analyze the values of *K* and *n* because of the fallacy of this approach: (1) despite the high *r*^2^, the values of the empirical constants *K* and *n* for each suspension depended on the shear rates used to fit the data, and (2) the meanings of *K* and *n* are not defined, except for systems with *n* = 1.

**Figure 6:**
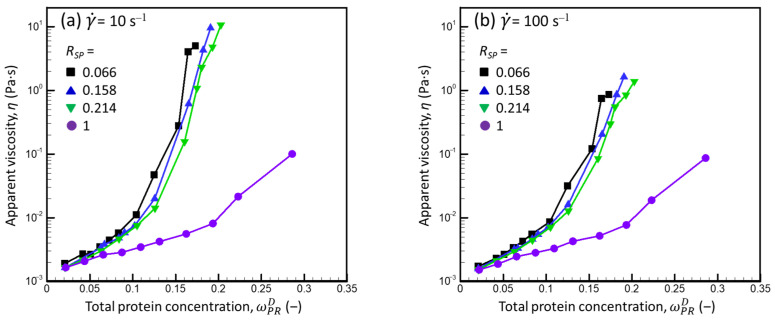
Apparent viscosity (*η*) measured at $\dot{\gamma }$ = 10 s^−1^ (**a**) and 100 s^−1^ (**b**) as a function of total protein concentration ($\omega_{PR}^{D}$) for dispersions with *R_SP_* = 0.066 (CNI, squares), 0.158 (triangles), 0.214 (inverted triangles), and 1 (WPI, circles).

To visualize the influence of the *R_SP_* on the rheological properties of mixed milk protein dispersions over the entire range of total protein concentrations, including the dispersions that did not reach limiting viscosity at the highest shear rate applied, we examined the dependencies of apparent viscosity on $\omega_{PR}^{D}$ for various $\dot{\gamma }$. Figure 6 presents the obtained dependencies for relatively low ($\dot{\gamma }$ = 10 s^−1^) and high ($\dot{\gamma }$ = 100 s^−1^) shear rates.

These results indicate the following:

(a) Regardless of the shear rate, for a given $\omega_{PR}^{D}$, the apparent viscosity decreased as the SP concentration in the dispersion increased. This behavior is expected: since SPs are about two orders of magnitude smaller than CMs, the increase in the *R_SP_* at a given $\omega_{PR}^{D}$ can be considered as replacing particles (CMs) with viscous liquid (WPs + SPs in PUF). The resulting increase in viscosity of the dm due to an increase in SP concentration was insufficient to compensate for the decrease in viscosity caused by removing CMs from the system.
(b) The apparent viscosity increased abruptly (much more than expected based on Equation (18)) when a certain protein concentration was exceeded. This behavior could be due to each CM being “caged” by its neighbors and subsequent collective movement in more concentrated dispersions [35]. This increase in *η* was observed at a higher $\omega_{PR}^{D}$ for dispersions with a higher *R_SP_*, which could be due to the decrease in the CM concentration as the *R_SP_* increased at a given $\omega_{PR}^{D}$.

For the highest $\omega_{PR}^{D}$ (0.173, 0.191, and 0.203 (g·g^−1^)), volume fractions of SP in the dm ($\varphi_{SP}^{dm}$) (calculated using Equation (A34)) reached 0.09, 0.24, and 0.34 in dispersions with *R_SP_* = 0.066, 0.158, and 0.214, respectively. It was thus necessary to verify whether the influence of the SPs was due only to an increase in the viscosity of the dm without a change in the hydrodynamic interactions between CMs.

It should be noted that some authors discussed the shear-flow properties of various dairy dispersions based on the dependencies estimated at a constant shear rate (e.g., the apparent or relative viscosity measured at a constant shear rate for different protein concentrations in the dispersion). This type of data analysis is not unconditional (i.e., it requires theoretical justification); for example, it can be valid for low- and high-shear-rate-limiting viscosities. However, in the current study, low and high-shear-rate-limiting viscosities were not reached for all dispersions.

Thus, we analyzed the data based on the theoretical approach of Krieger [36], who stated that when the dm has no specific influence on particle–particle interactions, there is a unique relation between the relative viscosity of dispersions with a given particle volume fraction and the shear stress applied (either between the relative viscosity and the product $\eta_{dm}\dot{\gamma }$), and this relation depends only on particle size (notably, it is independent of the viscosity of the dm). Thus, in the absence of specific interactions between the particles and molecules of a dm, the relative viscosity must be a function of only *τ* (either $\eta_{dm}\dot{\gamma }$, depending on which definition of the corresponding rheological states is correct).

Therefore, the relative viscosity of the mixed milk protein dispersions at a given $\eta_{dm}\dot{\gamma }$ was calculated as follows:

(a) For each mixed milk protein dispersion, $\varphi_{SP}^{dm}$ was calculated;
(b) For each $\dot{\gamma }$ applied for the measurement of the shear-flow curve of this dispersion, the apparent viscosity of its dm ($\eta_{dm}$) was obtained, assuming $\eta_{dm}\left(\varphi_{SP}^{dm},\dot{\gamma }\right)$ = $\eta_{WPI}$($\varphi_{SP}^{D}$,$\dot{\gamma }$), where $\eta_{WPI}$ is the apparent viscosity of the WPI dispersion with $\varphi_{SP}^{D}$ = $\varphi_{SP}^{dm}$;
(c) Values of $\eta_{WPI}$ at given $\varphi_{SP}^{D}$ and all required $\dot{\gamma }$ were calculated using point-by-point interpolation (for $\dot{\gamma }$ = 1–120 (s^−1^)) or extrapolation (for $\dot{\gamma }$ > 120 s^−1^ for the WPI dispersion that reached the high shear-rate-limiting viscosity at $\dot{\gamma }$ < 120 s^−1^) of the experimentally measured shear-flow curves of the WPI dispersions;
(d) Each value of $\eta_{dm}$ was used to calculate the parameter $\eta_{dm}\dot{\gamma }$ and the relative viscosity of the dispersion $\eta_{rdm}^{D}$; thus, the dependence of $\eta_{rdm}^{D}$ on $\eta_{dm}\dot{\gamma }$ was obtained;
(e) $\eta_{rdm}^{D}$ at a given $\eta_{dm}\dot{\gamma }$ was calculated using the power law point-by-point interpolation of the obtained dependence of $\eta_{rdm}^{D}$ on $\eta_{dm}\dot{\gamma }$;
(f) These calculations were performed for a range of $\eta_{dm}\dot{\gamma }$ of 0.005–1.4 (Pa).

**Figure 7:**
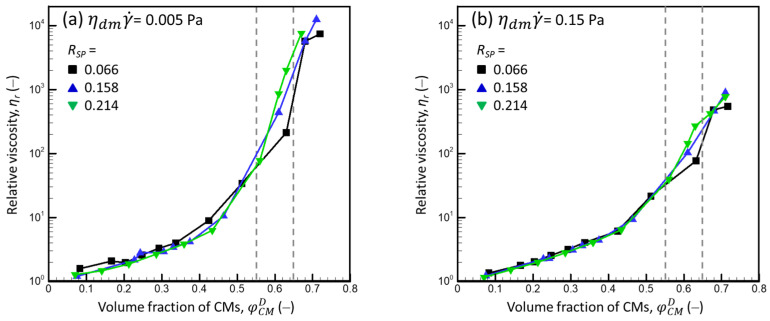
Relative viscosity of dispersion (*η_r_*) at $\eta_{dm}\dot{\gamma }$ of 0.005 Pa (**a**) and 0.15 Pa (**b**) as a function of the volume fraction of casein micelles (CMs) in dispersions with *R_SP_* = 0.066 (squares), 0.158 (triangles), and 0.214 (inverted triangles); dashed vertical lines correspond to $\varphi_{CM}^{D}$ = 0.55 and 0.65.

Figure 7 presents the dependencies of the relative viscosity of the mixed milk protein dispersions on the volume fraction of the CM obtained for low and high shear conditions: $\eta_{dm}\dot{\gamma }$ = 0.005 Pa ($\dot{\gamma }$ ≈ 1–10 (s^−1^)) and $\eta_{dm}\dot{\gamma }$ = 0.15 Pa ($\dot{\gamma }$ ≈ 20–150 (s^−1^)), respectively. We divided these dependencies into three parts: (1) *φ*_CM_ < 0.55, (2) *φ*_CM_ = 0.55–0.65, and (3) *φ*_CM_ > 0.65.

(1) At $\varphi_{CM}^{D}$ < 0.55 and high shear ($\eta_{dm}\dot{\gamma }$ ≥ 0.05 Pa) (e.g., at $\eta_{dm}\dot{\gamma }$ = 0.15 Pa, Figure 7b), the dependence of the relative viscosity on the CM volume fraction was practically independent of the *R_SP_* (even though, for a given $\varphi_{CM}^{D}$, the difference in SP concentration in the dm of dispersions with *R_SP_* = 0.214 was ca. 3.2 times larger than that with *R_SP_* = 0.066). However, at low shear ($\eta_{dm}\dot{\gamma }$ ≤ 0.01 Pa) (e.g., at $\eta_{dm}\dot{\gamma }$ = 0.005 Pa, Figure 7a), in the same concentration region, *η_r_* was slightly higher for dispersions with *R_SP_* = 0.066 than that with *R_SP_* = 0.158 or 0.214. Thus, at high shear, SPs had little influence on the hydrodynamic interactions between CMs when $\varphi_{CM}^{D}$ < 0.55, and they only increased the viscosity of the dm. Nonetheless, at low shear, SPs still had some influence on the CM interactions, which thus requires future study.

(2) In the range of the $\eta_{dm}\dot{\gamma }$ studied (0.005–1.4 Pa), *η_r_* increased sharply as *R_SP_* increased when $\varphi_{CM}^{D}$ > 0.55. This agrees with the results of Olivares et al. for the shear-flow behavior of the concentrated skim milk [23] and CNI dispersions [17]. According to [17,23]:

(a) Regardless of the shear conditions, the viscosity of the CNI dispersions [17] and concentrated skim milk [23] significantly increased when the critical volume fraction of CMs was exceeded (i.e., when the distance between the surfaces of the cores of CMs decreased to less than 10 nm, which is ca. twice the thickness of the *κ*-casein brush layer) due to the short-range repulsion between CMs;
(b) For a given distance between the surfaces of CM cores (<10 nm), the repulsion energy was much higher for concentrated milk than for the CNI dispersions;
(c) The higher repulsion energy between CMs in the concentrated milk did not impact the viscosity at low CM volume fractions.

Therefore, the sharp increase in viscosity at $\varphi_{CM}^{D}$ > 0.55 can be due to the contact and overlap of the *κ*-casein brush layers of CMs, and the increase in viscosity as the *R_SP_* increases could indicate that the short-range repulsion between CMs increases with the concentration of SPs.

(3) If the *κ*-casein layers of the CMs begin to touch at *φ_CM_* ≈ 0.55, increasing *φ_CM_* above 0.55 compresses the layers and causes them to overlap. Since the layers are ca. 5 nm thick, and CMs have a mean radius of ca. 90 nm, the value of *φ_CM_* at which CM cores begin to touch after the collapse of the *κ*-casein layer ($\varphi_{CM}$) was estimated as:

(19) $$\varphi_{CM}\approx 0.55{\left(\frac{90}{90-5}\right)}^{3}=0.65$$

According to Figure 7, where $\varphi_{CM}$ is shown by a dashed vertical line, regardless of the $\eta_{dm}\dot{\gamma }$, the *R_SP_* had less influence on *η_r_* when *φ_CM_* exceeded 0.65. This agrees with the hypothesis that a dispersion’s viscosity is determined by the mechanical properties of CM cores (and not by properties of the dm) when the CM cores are in direct contact.

It must be noted that the dependencies of *η_r_* on $\varphi_{CM}^{D}$ for different *R_SPs_* and $\eta_{dm}\dot{\gamma }$ were described relatively well by Equations (6) and (9) for the limited range of $\varphi_{CM}^{D}$ < 0.55. However, using two different equations to describe the data yielded different values for $\varphi_{m}$; also, it is difficult to conclude which equation is more suitable for the analysis (see the data and discussion in the Supplementary Materials). In addition, low and high shear-rate-limiting viscosities were not reached for most dispersions (Figure 4). Thus, our data were insufficient for further analysis of the influence of $\eta_{dm}\dot{\gamma }$ on the relative viscosity of mixed milk protein dispersions.

### 3.5. Oscillatory Measurements

**Figure 8:**
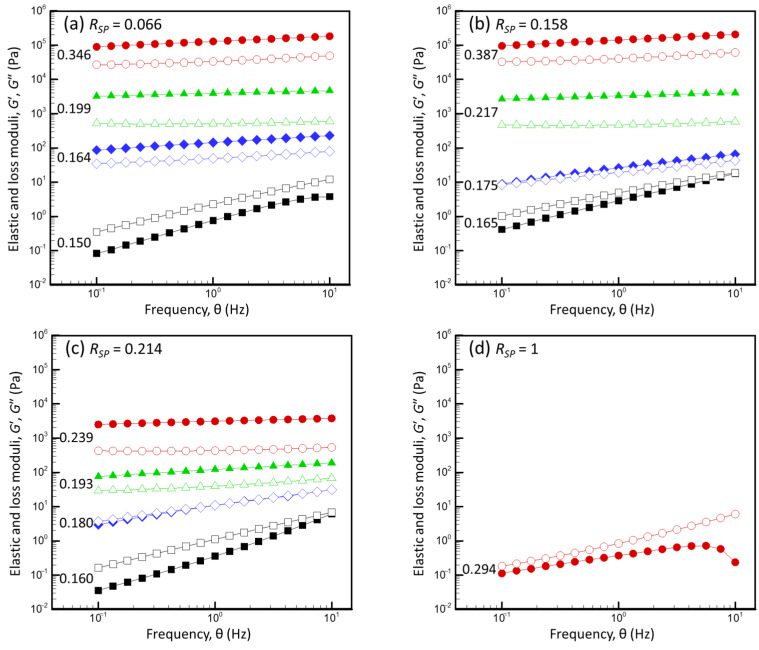
Elastic (*G*′) (solid symbols) and loss (*G*″) (open symbols) moduli as a function of frequency (*θ*) for mixed milk protein dispersions with *R_SP_* = 0.006 (CNI) (**a**), 0.158 (**b**), 0.214 (**c**), and 1 (WPI) (**d**). Symbols correspond to experimental data; total protein concentrations ($\omega_{PR}^{D}$) are shown next to the curves.

Figure 8 presents examples of the experimentally measured dependencies of the elastic modulus (*G*′) and the loss modulus (*G*″) on the frequency (*θ*) for dispersions with different total protein concentrations ($\omega_{PR}^{D}$) and SP weight fractions (*R_SP_*).

For all *R_SP_*, *G*′ < *G*″ and *G*′ strongly depended on *θ* at low $\omega_{PR}^{D}$. Thus, the low-concentration dispersions behaved like viscoelastic liquids, whereas the higher-concentration dispersions behaved like viscoelastic solids with *G*′ > *G*″ and demonstrated a moderate dependency of *G*′ on *θ*. Only the WPI dispersion (*R_SP_* = 1) remained liquid at the highest studied concentration (0.294). For the range of frequency of 0.1–1.0 Hz, all dependencies of *G*′ on *θ* were described relatively well by the power law equation:

(20) $$G\;\propto \;\theta^{m}$$

where *m* is a fitting parameter.

**Figure 9:**
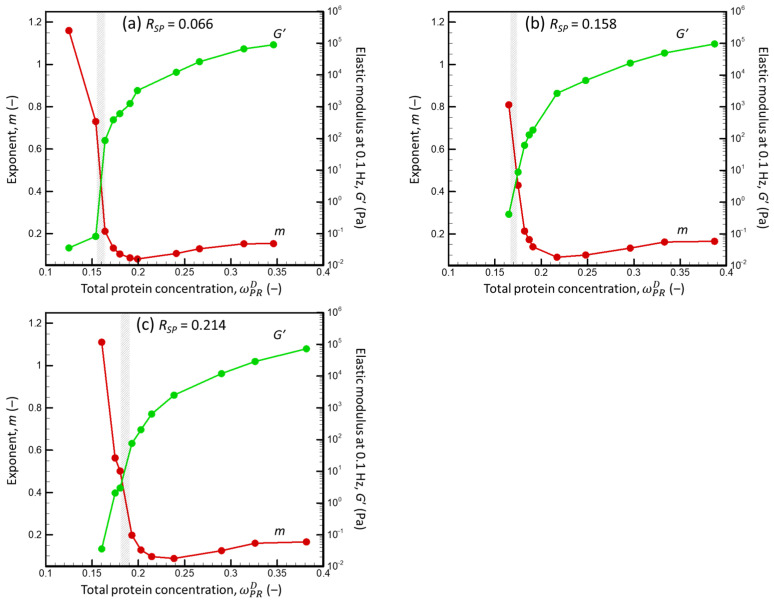
Elastic modulus *G*′ (measured at a frequency (θ) of 0.1 Hz) and exponent *m* (estimated using Equation (20)) for mixed milk protein dispersions prepared by osmotic compression for *R_SP_* = 0.066 (CNI) (**a**), 0.158 (**b**), and 0.214 (**c**). The left and right edges of shaded zones correspond to dispersions with liquid-like (*G*′ < *G*″) or solid-like (*G*′ > *G*″) behavior, respectively, in the range of θ of 0.1–10.0 Hz.

To define the concentrations, where the behavior of dispersions changed from liquid-like to solid-like (i.e., the sol–gel transition concentration ($\omega_{PR}^{D,sg}$)), we examined *G*′ at θ = 0.1 Hz and *m* as a function of $\omega_{PR}^{D}$ (Figure 9).

Regardless of the *R_SP_* (0.066–0.214), *G*′ increased sharply, and *m* decreased sharply as the $\omega_{PR}^{D}$ increased in the narrow range of total protein concentrations (Δ$\omega_{PR}^{D}$ around 0.01), where *G*′ ∝ ($\omega_{PR}^{D}$)*^k^* had an exponent *k* of 40–100, while above the critical concentration region, *k* is ca. 5. The sol–gel transition occurred at higher $\omega_{PR}^{D}$ in suspensions with a higher *R_SP_*. In addition, far above the sol–gel transition point, both elastic and loss moduli decreased significantly as the *R_SP_* increased, which implies that for a given total protein concentration, gels are less rigid but also less viscous at a higher *R_SP_*.

**Figure 10:**
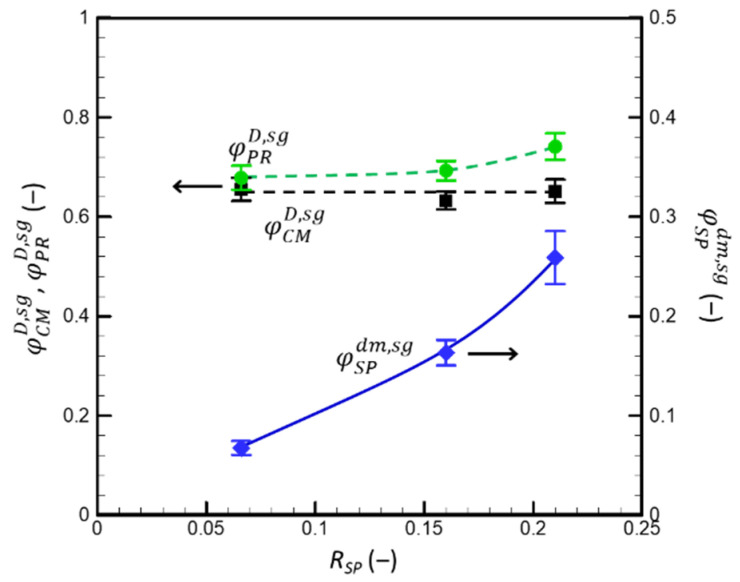
Composition of dispersions in the sol–gel transition region as a function of the relative concentration of serum proteins (*R_SP_*): particle volume fraction of casein micelles (CMs) in the dispersion ($\varphi_{CM}^{D,sg}$) (squares), the volume fraction of proteins in the dispersion ($\varphi_{PR}^{D,sg}$) (circles), and volume fraction of serum proteins in the dm ($\varphi_{SP}^{dm,sg}$) (diamonds). Symbols correspond to the middle of the sol–gel transition region, while error bars indicate its upper and lower limits (shaded zones in Figure 9). The dashed horizontal line indicates the mean $\varphi_{CM}^{D,sg}$ (0.65).

To discuss the roles of SPs and CMs in gel formation, the particle volume fraction of the CMs ($\varphi_{CM}^{D}$) and total proteins ($\varphi_{PR}^{D}$), as well as the volume fraction of the SPs in the dm ($\varphi_{SP}^{dm}$) in the sol–gel transition region (determined as shown in Figure 9) were calculated for dispersions with different *R_SP_*, assuming a *v_CM_* of 4.15 mL·g^−1^ and *v_SP_* of 2.09 mL·g^−1^ (Figure 10).

At the point of gel formation, although the SP concentration in the dm increased significantly as the *R_SP_* increased, the CM volume fraction ($\varphi_{CM}^{D,sg}$) remained nearly constant (ca. 0.65). This value agrees with the critical CM volume fraction ($\varphi_{CM}$) of ca. 0.65, estimated from the shear-flow analysis, at which the CM cores came into direct contact (Equation (19)). Thus, the sol–gel transition in mixed milk protein dispersions can be explained by the direct contact between CM cores, which is defined the micelle shape and polydispersity and does not depend on the presence of soluble compounds such as SPs in the dm (e.g., in pores between the touching micelles). As expected, the presence of SPs in the dm increased the volume fraction of the total proteins in the system at a given $\varphi_{CM}^{D,sg}$.

Mixed dispersions had some liquid-like behavior even above the sol–gel transition observed via oscillatory measurements ($\varphi_{CM}^{D}$ > 0.65, Figure 7). This behavior is common for soft particles that can be deformed by applying shear and has been observed for dispersions of CMs [6].

## 4. Discussion

### 4.1. Serum Protein Distribution in Mixed Milk Protein Dispersions

There was good correspondence between the measured and predicted total DM content and viscosity of the dm of mixed milk protein dispersions with component concentrations ranging from $\omega_{CN}^{D}$ = 0.078, $\omega_{SP}^{D}$ = 0.005 and $\omega_{CN}^{D}$ = 0.117, and $\omega_{SP}^{D}$ = 0.008 (*R_SP_* = 0.066) to $\omega_{CN}^{D}$ = 0.100 and $\omega_{SP}^{D}$ = 0.026 (*R_SP_* = 0.021) when the *v_CM_* and *f_SP_* equaled 4.15 mL·g^−1^ and 0, respectively (Figure 1 and Figure 3). Because *f_SP_* ≡ $\left(m_{SP}^{CM}/m_{w,CM}^{D}\right)/\left(m_{SP}^{dm}/m_{w,dm}^{D}\right)$ (Equation (A2)), *f_SP_* = 0 when $m_{SP}^{CM}$ = 0, which implies that CMs do not absorb SPs (in the ranges of the $\omega_{CN}^{D}$ and $\omega_{SP}^{D}$ studied, and when the volume of the dm absorbed by CMs is defined by a *v_CM_* of 4.15 mL·g^−1^).

However, Peixoto et al. [4] reported partition coefficients for the WPs (*K_SP_*) in mixed dispersions of SPs and CMs of 4.0–4.5 for *α*-lactalbumin and 6.0–6.5 for *β*-lactoglobulin, where *K_SP_* ≡ $\left(m_{SP}^{CM}/V_{CM}^{D}\right)/\left(m_{SP}^{dm}/V_{dm}^{D}\right)$, which indicated that CMs strongly absorbed SPs. These values were calculated by analyzing the fluorescence recovery after photobleaching CM dispersions with casein concentrations of 0.020–0.250 g·mL^−1^, with an initial *R_SP_* of ca. 0.05, along with 0.0005 or 0.005 g·mL^−1^ of an added probe (i.e., labeled *α*-lactalbumin or *β*-lactoglobulin). Thus, the dispersions had component concentrations that ranged from $\omega_{CN}^{D}$ = 0.020 and $\omega_{SP}^{D}$ ≈ 0.0016–0.0061 to $\omega_{CN}^{D}$ = 0.250 and $\omega_{SP}^{D}$ ≈ 0.014–0.018. Thus, the results of [4] can be compared directly to those of the present study for the CNI dispersions (*R_SP_* = 0.066). In contrast, comparing the results for *R_SP_* = 0.158 and 0.214 requires assuming that *K_SP_* remains constant at a higher *R_SP_* (0.158–0.214 in the present study vs. 0.05–0.10 in [4]) for a given CN concentration of ca. 0.1 g·mL^−1^.

**Figure 11:**
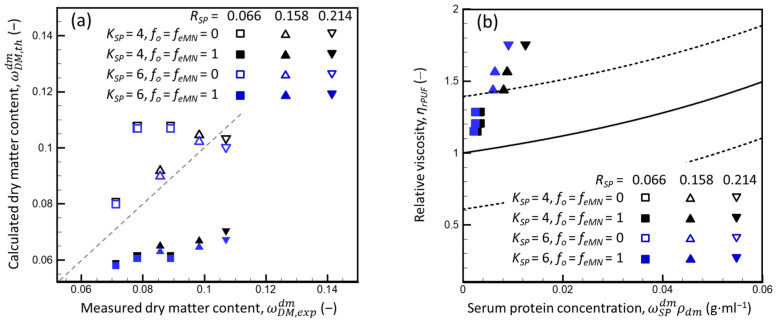
(**a**) Theoretical dry matter (DM) content of the dispersion medium ($\omega_{DM,th}^{dm}$) (calculated for the assumed values of *K_SP_*, *f_o_*, and *f_eMN_* listed) vs. measured total DM content of supernatants ($\omega_{DM,exp}^{dm}$) of mixed milk protein dispersions. Data for suspensions with *R_SP_* = 0.066 and $\omega_{PR}^{D}$ = 0.083 and 0.125 (squares), *R_PS_* = 0.158 and $\omega_{PR}^{D}$ = 0.105 and 0.126 (triangles), and *R_SP_* = 0.214 and $\omega_{PR}^{D}$ = 0.126 (inverted triangles). The dashed line is the 1:1 line. The DM content at the origin is that of PUF (0.0521). (**b**) Relative viscosity ($\eta_{rPUF}$) at a shear rate of 100 s^−1^ as a function of the concentration of serum protein in supernatants ($\omega_{SP}^{dm}\rho_{D}$) (calculated for the assumed values of *K_SP_*, *f_o_*, and *f_eMN_* listed); data for dispersions with *R_SP_* = 0.066 and $\omega_{PR}^{D}$ = 0.083 and 0.125 (squares), *R_SP_* = 0.158 and $\omega_{PR}^{D}$ = 0.105 and 0.126 (triangles), and *R_SP_* = 0.214 and $\omega_{PR}^{D}$ = 0.126 (inverted triangles); solid curve corresponds to data described by Lee’s equation, while dashed curves correspond to lower and upper limits of the 95% prediction interval.

Using the relation between *K_SP_* and *f_SP_*

(21) $$f_{SP}=K_{SP}\frac{v_{CM\omega_{CN}^{D}}}{\rho_{D}^{-1}-v_{CM\omega_{CN}^{D}}}\left(\frac{\omega_{w}^{D}}{\omega_{w,CM}^{D}}-1\right)$$

and assuming different combinations of *K_SP_* = 4 or 6 with *f_o_* = *f_eMN_* = 0 or 1, the DM content and SP concentration in the supernatants were estimated for the mixed milk protein dispersions used in the present study. Figure 11a,b are presented in the same way as in Figure 1 and Figure 3b, respectively.

The $\omega_{DM,th}^{dm}$ estimated based on the assumed values *K_SP_* = 4 or 6 and *fo* = *f_eMN_* = 1 were much lower than those measured experimentally $\omega_{DM,exp}^{dm}$. In contrast, the $\omega_{DM,th}^{dm}$ estimated for *K_SP_* = 4 or 6 and *fo* = *f_eMN_* = 0 were either higher (*R_SP_* = 0.066 and 0.158) or lower (*R_SP_* = 0.214) (Figure 11a). In addition, the dependencies of relative viscosity of the supernatants on $\omega_{SP}^{dm}\rho_{D}$ calculated for *K_SP_* = 4 and 6 differed greatly from that expected for the SP dispersions in the PUF (Figure 11b) and were nearly insensitive to *f_o_* and *f_eMN_*. Thus, the results for the SP distribution between the dm and CMs in mixed dispersions of SPs and CMs did not agree with those of [4]. This difference could be due to differences in the CNI and WPI used in the two studies (e.g., thermal treatment used for milk, and the drying protocol used for the CNI and WPI) or the experimental methods and data analysis (the in situ analysis followed by modeling by [4], and ultracentrifugation followed by the DM analysis in the present study). However, additional research is needed to explain this disagreement.

### 4.2. Rheological Properties of the Dispersion Medium

According to the results presented in Figure 3b, the influence of the SP concentration on high shear-rate-limiting viscosity of the dm of mixed milk protein dispersions can be estimated using Lee’s equation and an SP voluminosity (*v_SP_*) of 2.09 mL·g^−1^, at least for $\omega_{SP}^{dm}\rho_{D}$ ≤ 0.05. For the SP concentrations studied, the viscosities of the serums were similar to those of the WPI dispersions (Figure 3a). Thus, it can be expected that this equation remains valid for the more concentrated serums with $\omega_{SP}^{dm}\rho_{D}$ ≤ 0.21. Finally, in the range of the $\omega_{SP}^{dm}\rho_{D}$ studied, SPs have a non-negligible influence on the viscosity of the dm, which must be considered when analyzing the rheological properties of mixed protein dispersions.

### 4.3. Rheological Properties of Mixed Protein Dispersions, Role of R_SP_

According to Figure 5b, the high shear-rate-limiting viscosity of the mixed protein dispersions relative to the viscosity of the serum can be described by Lee’s equation using a CM voluminosity (*v_CM_*) of 4.15 mL·g^−1^ (at least for *φ_CM_* ≤ 0.38 and *R_SP_* ≤ 0.214). Thus, within the range of the *φ_CM_* and *R_SP_* studied, the rheological behavior of CMs can be described as that of hard spheres, with a hydrodynamic radius corresponding to the value of the *v_CM_* estimated. This implies that SPs (at least at $\omega_{SP}^{dm}$ ≤ 0.036) did not influence the CM voluminosity, although the presence of SPs increased the viscosity of the dm. Interestingly, the *v_CM_* estimated via the shear-flow analysis agreed with what was required to explain the independent experimental data (i.e., the DM content and viscosity of serums).

When *φ_CM_* > 0.5, the relative viscosity of the mixed dispersions was not described adequately by Lee’s equation (which does not apply to high particle volume fractions) or the equations of Quemada or Mendoza–Santamaria-Holek (Figures S4 and S5 in Supplementary Materials). The relative viscosity increased significantly as the *R_SP_* increased when *φ_CM_* > 0.5 and became less sensitive to the *R_SP_* when *φ_CM_* > 0.65. In agreement with the findings from previous studies [12,17,23], this could be due to a large increase in repulsion between CMs in the presence of SPs when the *κ*-casein brush layers begin to overlap (*φ_CM_* > 0.5). In addition, the influence of the SPs decreased in the jammed dispersions, in which CM cores come into direct contact (*φ_CM_* > 0.65), and viscosity is determined mainly by CM deformation. It must be noted that this explanation requires that contact between the micelles (including the *κ*-casein brush layers) occurs at a *φ_CM_* below the maximum volume fraction of random close packing for the monodisperse spheres (*φ_rcp_* ≈ 0.64). If this hypothesis is correct, it implies that:

(a) either the *κ*-casein brush layers have little influence on the hydrodynamic behavior of CMs in diluted dispersions (described by Lee’s equation with a *v_CM_* of 4.15 mL·g^−1^)
(b) or the shear applied to the concentrated dispersions assembles the CMs (in agreement with the hypothesis of Noebel et al. [11,15]).

The latter explanation is more likely; however, the present study did not produce enough data for dispersions with *φ_CM_* > 0.5 to draw conclusions about the structure of mixed dispersions under applied shear.

### 4.4. The Sol–Gel Transition

The sol–gel transition volume fraction of the CMs (ca. 0.65) observed via oscillatory measurements was nearly independent of the SP concentration in the mixed dispersions (*R_SP_* = 0.066–0.214, Figure 10). This value is close to that reported in previous studies for the CNI and concentrated milk dispersions (0.64–0.78) (estimated by fitting shear-rate data or observed via oscillatory measurements [6,10,11,12,15,19]) but much lower than that estimated ones by Corredig and co-workers by fitting high-shear-rate data for concentrated milk [20,21,22]. We do not know whether this difference is due to differences in the data analysis and/or sample sources. However, the CM volume fraction of 0.65, obtained from the oscillatory measurements, is consistent with that obtained from the shear-flow analysis (Figure 7, Equation (19)).

## 5. Conclusions

CMs were not observed to absorb SPs in mixed dispersions of SPs, and CMs with $\omega_{CN}^{D}$ = 0.078–0.117 and *R_SP_* = 0.066–0.214.

The high shear-rate-limiting viscosity of the dm was described well by Lee’s equation using an SP voluminosity of 2.09 mL·g^−1^. Lee’s equation was also valid for describing the high shear-rate-limiting viscosity of SP dispersions with $\omega_{SP}^{D}$ ≤ 0.05.

When *φ_CM_* ≤ 0.38, the high shear-rate-limiting viscosity of mixed protein dispersions relative to the viscosity of serum was described well by Lee’s equation with a CM voluminosity of 4.15 mL·g^−1^; the latter was constant in the range of *R_SP_* = 0.066–0.214.

At *R_SP_* = 0.066–0.214, the sol–gel transition volume fraction of CMs was nearly constant and equal to 0.65.

## Figures and Tables

**Table 1:** Weight fractions (*ω*) of components in CNI, WPI, and PUF (in wt.%).

	$\omega_{DM}$	$\omega_{MN}$	$\omega_{NPN}$	$\omega_{CN}$	$\omega_{mCN}$	$\omega_{℘}$	$\omega_{SP}$	$\omega_{PR}$	$\omega_{o}$
CNI	93.76	8.04	1.04	77.92	77.92	5.46	5.46	83.38	2.34
WPI	93.81	1.78	0.84	7.92	0	79.53	87.45	87.45	4.58
PUF	5.21	0.50						0	4.71

DM—dry matter, MN—minerals, NPN—non-protein-nitrogen compounds, CN—all caseins, mCN—micellar caseins, WP—whey proteins, SP—serum proteins, PR—total proteins, o—low-molecular-weight organic compounds.

**Table 2:** Sets of assumptions about the ability of CMs to absorb solutes: combinations of *f_i_* values for serum proteins *f_SP_*, organic low-molecular-weight compounds *f_o_*, and soluble (“extra”) mineral compounds *f_eMN_*.

Set of Assumptions:	A	B	C	D	E	F	G	H
*f_SP_* =	0	0	0	0	1	1	1	1
*f_o_* =	0	0	1	1	1	0	1	0
*f_eMN_* =	0	1	0	1	1	0	0	1

## Data Availability

The original contributions presented in the study are included in the article/Supplementary Materials, further inquiries can be directed to the corresponding author (M.L.).

## Supplementary Materials

The supporting information can be downloaded at https://www.mdpi.com/article/10.3390/foods13213480/s1, Figure S1: DM of dm; Figures S2 and S3: *η* of WPI dispersions; Figure S4: *η* of mixed milk protein dispersions; Figure S5: values of *φ_m_*; Table S1: values of *K* and *n*.

## Nomenclature

**Abbreviations**	
aMN	mineral compounds associated with casein micelle structure
CD	“concentrated/compressed” dispersion prepared by osmotic compression
CM	casein micelle
CN	casein
mCN	micellar casein
sCN	serum casein
CNI	native phosphocaseinate isolate
D	dispersion
dm	dispersion medium
DM	dry matter
eMN	“extra” (solubilized) mineral compounds
exp	experimental
LD	“liquid” dispersion prepared without osmotic compression
MN	minerals
NCN	non-casein-nitrogen containing compounds
NPN	non-protein-nitrogen containing compounds
o	low-molecular-weight organic compounds
PEG	polyethylene glycol
PR	total proteins
PUF	permeate produced by ultrafiltration of skim milk
sg	sol–gel point
SP	serum protein
th	theoretical
TN	total-nitrogen-containing compounds
w	water
WP	whey protein
WPI	whey protein isolate
**Latin letters**	
*a_DM_*	parameter defined by Equation (A17) (–)
*a_o_*	parameter defined by Equation (A18) (–)
*a_H_*	hydrodynamic radius of CM (nm)
*C*	parameter defined by Equation (10) (–)
*f_i_*	relative solubility of component *i* defined by Equation (A2) (–)
*G*′	elastic modulus (Pa)
*G*″	loss modulus (Pa)
*K*	empirical parameter of Equation (13) (Pa·s^1+*n*^)
*m*	parameter of Equation (22) (–)
*m_i_*	weight of *i* (g)
$m_{i}^{j}$	weight of *i* in *j* (g)
$m_{i,z}^{j}$	weight of *i* belonging to *z* in *j* (g)
*n*	empirical parameter of Equation (13) (–)
*q*	parameter in Equation (7) (–)
*R_aMN_*	parameter defined by Equation (A1) (–)
*R_SP_*	weight fraction of serum proteins in total proteins (–)
*R_w_*	parameter defined by Equation (A22) (–)
*U*	energy of interparticle repulsion (J)
*V_i_*	volume of *i* (mL)
$V_{i}^{j}$	volume of *i* in *j* (mL)
$V_{i,z}^{j}$	volume of *i* belonging to *z* in *j* (mL)
*v_CM_*	voluminosity of casein micelles (mL·g^−1^)
*v_SP_*	voluminosity of serum proteins (mL·g^−1^)
**Greek letters**	
$\dot{\gamma }$	shear rate (s^−1^)
$\dot{\gamma_{c}}$	characteristic shear rate (s^−1^)
*η*	apparent viscosity (Pa·s)
*η*_0_	zero (low)-shear-rate-limiting viscosity (Pa·s)
*η_∞_*	infinite (high)-shear-rate-limiting viscosity (Pa·s)
*η_i_*	apparent viscosity of *i* (Pa·s)
*η_r_*	relative viscosity (Pa·s)
*η_r,_*_0_	relative zero (low)-shear-rate-limiting viscosity (Pa·s)
$\eta_{ri}^{j}$	viscosity of *j* relative to viscosity of *i* (–)
*η_rPUF_*	viscosity of dispersion relative to viscosity of PUF (–)
*η_rPUF,0_*	zero (low)-shear-rate-limiting viscosity of dispersion relative to viscosity of PUF (–)
*η_rPUF,∞_*	infinite (high)-shear-rate-limiting viscosity of dispersion relative to viscosity of PUF (–)
[*η*]	intrinsic viscosity (–)
*θ*	frequency (Hz)
*π*	osmotic pressure (Pa)
*ρ_i_*	density of *i* (g·mL^−1^)
*τ*	applied shear stress (Pa)
*τ_c_*	characteristic shear stress (Pa)
*φ*	particle volume fraction (–)
*φ*_0_	parameter of Equation (6) when applied in the equation of Mendoza–Santamaria-Holek for zero-shear-rate-limiting viscosity (–)
*φ*_∞_	parameter of Equation (6) when applied in the equation of Mendoza–Santamaria-Holek for infinite-shear-rate-limiting viscosity (–)
*φ_i_*	particle volume fraction of *i* (–)
$\varphi_{i}^{j}$	volume fraction of *i* in *j* (–)
$\varphi_{i,z}^{j}$	volume fraction of *i* belonging to *z* in *j* (–)
$\varphi_{CM}$	critical volume fraction of casein micelles (–)
*φ_m_*	critical volume fraction of particles at which the viscosity of dispersion diverges (–)
*φ_CMmax,_*_0_	maximum volume fraction of casein micelles at which the relative zero (low)-shear-rate-limiting viscosity diverges (–)
*φ_CMmax,_*_∞_	maximum volume fraction of casein micelles at which the relative infinite (high)-shear-rate-limiting viscosity diverges (–)
*ω_i_*	weight fraction of component *i* (–)
$\omega_{i}^{j}$	weight fraction of component *i* in *j* (–)
$\omega_{i,z}^{j}$	weight fraction of *i* belonging to *z* in *j* (–)

## Appendix A. Composition of Dispersions and Component Distribution Between CMs and the Dispersion Medium

### Appendix A.1. General Consideration

Below, the “liquid” dispersions prepared by dispersing CNI and/or WPI powder in PUF and “compressed” dispersions prepared by dispersing powder followed by osmotic compression are denoted by superscripts LD and CD, respectively. The superscript D is used when the dispersion type is not relevant (e.g., the equation is valid for both LD and CD).

All studied dispersions (including those prepared via osmotic compression) contained the same components as CNI, WPI, and PUF: micellar caseins (mCNs), serum proteins (SPs), low-molecular-weight organic compounds (o), mineral compounds (MN), and water (w). Since all experiments were conducted at 20 °C, we assumed that all caseins from the CNI were associated with CMs [37]. Thus, all dispersions studied were assumed to be CMs (disperse phase) surrounded by a serum/dispersion medium (dm).

In addition to casein molecules, CMs contain associated mineral compounds (K^+^, Na^+^, Ca^2+^, Mg^2+^, and colloidal calcium phosphate) (aMN). The total weight of aMN per weight of caseins in the micelles (so, the weight of micellar caseins in dispersions) (*R_aMN_*) was calculated as

(A1) $$R_{aMN}=\omega_{aMN}^{CM}/\omega_{mCN}^{CM}=\omega_{aMN}^{D}/\omega_{mCN}^{D}$$

where $\omega_{aMN}^{CM}$ and $\omega_{mCN}^{CM}$ are the weight fractions of aMN and mCNs in CMs, respectively, and $\omega_{aMN}^{D}$ and $\omega_{mCN}^{D}$ are the weight fractions of aMN and mCNs in the dispersion, respectively.

According to [38], in addition to the caseins, 1 g of dry isolated CMs contains 0.074 g of aMN: 0.059 g of calcium phosphate nanocrystals and 0.015 g of exchangeable inorganic cations. Thus, the total content of the mineral compounds associated with caseins in CMs can be estimated as *R_aMN_* = $0.074/\left(1-0.074\right)$ = 0.080. We assumed that *R_aMN_* is a structural property of CMs; thus, a *R_aMN_* of 0.080 is independent of the composition, concentration, or preparation method of the dispersions.

The difference between the total mineral compounds in the dispersions and the mineral compounds associated with CMs was considered as “extra” mineral compounds (eMN)*,* which came mainly from PUF, as well as from residual soluble mineral compounds in the CNI and WPI. Using Equation (A1), the weight fraction of eMN in the dispersion ($\omega_{eMN}^{D}$) was calculated as $\omega_{eMN}^{D}=\omega_{MN}^{CM}-R_{aMN}\omega_{mCN}^{CM}$, where $\omega_{MN}^{CM}$ is the weight fraction of total mineral compounds.

In aqueous dispersions, CMs are highly porous particles that swell when they absorb water (or dm). The swelling of CMs increases the volume of micelles (compared to that of dry micelles). The extent of micelle swelling is characterized by the CM voluminosity (*v_CM_*) (defined by Equation (1)).

The high porosity of swollen CMs can enable them to absorb low- and high-molecular-weight compounds [4]. However, in general, it is unknown whether CMs can absorb SPs, o, and eMN from the dm (or what the distribution of SPs, o, and eMN is between the CMs and dm). To quantify the distribution of solute *i* (where *i* is SPs, o, or eMN) between CMs and dm in the dispersions, we introduced parameter *f_i_*, which is the relative weight content of solute *i* in the water absorbed by the CMs compared to that in the water remaining in the dm:

(A2) $$f_{i}=\frac{m_{i}^{CM}}{m_{w}^{CM}}/\frac{m_{i}^{dm}}{m_{w}^{dm}}=\frac{\omega_{i,CM}^{D}}{\omega_{w,CM}^{D}}/\frac{\omega_{i,dm}^{D}}{\omega_{w,dm}^{D}}$$

where $m_{i}^{CM}$ and $m_{w}^{CM}$ are the weights of component *i* and the water present in (absorbed by) CMs, respectively; $m_{i}^{dm}$ and $m_{w}^{dm}$ are the weights of the same component *i* and the water in the dm, respectively; $\omega_{i,CM}^{D}$ and $\omega_{w,CM}^{D}$ are the weight fractions in the dispersion of component *i* and the water present in CMs, respectively, and $\omega_{i,dm}^{D}$ and $\omega_{w,dm}^{D}$ are the weight fractions in the dispersion of component *i* and the water present in the dm, respectively.

We assumed that *f_SP_*, *f_o_*, and *f_eMN_* defined by Equation (A2) are an inherent property of the system: the ability of the water absorbed by CMs to dissolve SPs, o, and eMN compared to that of water in the dm. Thus, we assumed that *f_SP_*, *f_o_*, and *f_eMN_* were independent of the dispersion composition and preparation method (“liquid” (LD) or “compressed” (CD) dispersion). In general, the *f_i_* values must be determined experimentally.

### Appendix A.2. Determining fi for Dispersions with Known Total Composition

Considering the mass balances for component *i* and for water in the dispersion

(A3) $$\omega_{i}^{D}=\omega_{i,CM}^{D}+\omega_{i,dm}^{D}$$

(A4) $$\omega_{w}^{D}=\omega_{w,CM}^{D}+\omega_{w,dm}^{D}$$

(where $\omega_{i}^{D}$ and $\omega_{w}^{D}$ are the weight fractions of *i* and water in the dispersions, i.e., the total composition of the dispersion), one can obtain

(A5) $$\omega_{i,CM}^{D}=\frac{f_{i}\omega_{i}^{D}\omega_{w,CM}^{D}}{\omega_{w}^{D}-\left(1-f_{i}\right)\omega_{w,CM}^{D}}$$

The volume of the swelled CMs in the dispersion ($V_{CM}^{D}$) is calculated as the sum of the volumes of its components

(A6) $$V_{CM}^{D}=V_{mCN}^{D}+V_{aMN}^{D}+V_{w,CM}^{D}+\sum_{i}V_{i,CM}^{D}$$

where $V_{mCN}^{D}$, $V_{aMN}^{D}$, and $V_{w,CM}^{D}$ are the volumes in the dispersion of the mCNs, aMN, and water absorbed by CMs, respectively, and $V_{i,CM}^{D}$ is the volume of each component *i* (SPs, o, and eMN) in the dispersion, which is absorbed by swollen CMs, and its quantity in the dispersion is defined by Equation (A5).

s on the specific weight of the CM components (*ρ_i_*), one can calculate $\nu_{CM}$ from Equations (A1), (1), (A5) and (A6):

(A7) $$v_{CM}=\frac{1}{\rho_{mCN}}+\frac{R_{aMN}}{\rho_{MN}}+\frac{1}{\rho_{w}}\frac{\omega_{w,CM}^{D}}{\omega_{mCN}^{D}}+\frac{1}{\omega_{mCN}^{D}}\sum_{i}\frac{1}{\rho_{i}}\frac{f_{i}\omega_{i}^{D}\omega_{w,CM}^{D}}{\omega_{w}^{D}-\left(1-f_{i}\right)\omega_{w,CM}^{D}}$$

where *i* is summed for the SPs, o, and eMN.

When the total composition of the dispersion is known ($\omega_{mCN}^{D}$, $\omega_{w}^{D}$, and all $\omega_{i}^{D}$ values are known), Equation (A7) can be solved (i.e., $\omega_{w,CM}^{D}$ can be found) for the known values of *f_i_*.

Once $\omega_{w,CM}^{D}$ is determined, the weight fractions of absorbed SPs, o, and eMN in the dispersion (i.e., all $\omega_{i,CM}^{D}$) are calculated using Equation (A5). Further,

(a) The weight fractions of all non-absorbed components (solutes and water that constitute the dm) in the dispersion are calculated based on the mass balance (Equations (A3) and (A4));
(b) The weight fraction of the dm in the dispersion is calculated as the sum of the weight fractions of its components:
  (A8) $$\omega_{dm}^{D}=\omega_{w,dm}^{D}+\sum_{i}\omega_{i,dm}^{D}$$
  where *i* = SPs, o, and eMN;
(c) The weight fraction of solute *i* in the dm is calculated as
  (A9) $$\omega_{i}^{dm}=\omega_{i,dm}^{D}/\omega_{dm}^{D}$$
(d) The total dry matter content in the dm is calculated as
  (A10) $$\omega_{DM}^{dm}=\sum_{i}\omega_{i}^{dm}$$
  where *i* = SPs, o, and eMN.

For *f_SP_* = 0, *f_o_* = 1, and *f_eMN_* = 0, Equation (A7) is simplified to

(A11) $$\omega_{w,CM}^{D}=\omega_{mCN}^{D}\frac{v_{CM}-\rho_{mCN}^{-1}-\rho_{mn}^{-1}R_{aMN}}{\rho_{w}^{-1}+\rho_{o}^{-1}\left(\omega_{o}^{D}/\omega_{w}^{D}\right)}$$

### Appendix A.3. Total Composition of Dispersions Prepared via CNI and WPI Dispersed in PUF

The total compositions (weight fractions of components) of the milk protein dispersions prepared via CNI and/or WPI powders mixed with PUF (i.e., the composition of LD) were calculated knowing the quantities and compositions of the CNI, WPI, and PUF:

(A12) $$\omega_{i}^{LD}=m_{i}^{LD}/m_{LD}$$

where *m_LD_* is the weight of the dispersion and $m_{i}^{LD}$ is the weight of component *i* in the dispersion (*i* stands for mCN, SPs, o, MN, or w). For the known values of *f_i_* (*i* = SPs, o, and eMN), the distribution of the components between the CMs and dm was calculated as explained above.

### Appendix A.4. Composition of CM-Containing Dispersions Prepared via Osmotic Compression

The molecular weight cut-off of the dialysis bag material was high enough to allow low-molecular-weight organic and mineral compounds, as well as water, to penetrate the membrane and reach an equilibrium between the CD and stressing polymer solutions. We assumed that the equilibrium concentrations of o and eMN in the dm of the CD and in the stressing polymer solutions were not influenced by the presence of CMs and SPs (in the CD) or PEG (in stressing polymer solutions). In other words, the relative content of o and eMN in the dm of the CD was assumed to equal that in the dm of the stressing polymer solutions (i.e., in PUF):

(A13) $$m_{o,dm}^{CD}/m_{w,dm}^{CD}=m_{o}^{PUF}/m_{w}^{PUF}\;\mathrm{and}\;\omega_{o,dm}^{CD}/\omega_{w,dm}^{CD}=\omega_{o}^{PUF}/\omega_{w}^{PUF}$$

(A14) $$m_{eMN,dm}^{CD}/m_{w,dm}^{CD}=m_{MN}^{PUF}/m_{w}^{PUF}\;\mathrm{and}\;\omega_{eMN,dm}^{CD}/\omega_{w,dm}^{CD}=\omega_{MN}^{PUF}/\omega_{w}^{PUF}$$

where $m_{w,dm}^{CD}$, $m_{o,dm}^{CD}$ and $m_{eMN,dm}^{CD}$ are the weights of water, o, and eMN, respectively, in the dm of the CD; $m_{w}^{PUF}$, $m_{o}^{PUF}$, and $m_{MN}^{PUF}$ are the weights of water, o, and MN, respectively, in PUF; $\omega_{w,dm}^{CD}$, $\omega_{o,dm}^{CD}$, and $\omega_{eMN,dm}^{CD}$ are the weight fractions of water, o, and eMN, respectively, in the dm of the CD; and $\omega_{w}^{PUF}$, $\omega_{o}^{PUF}$, and $\omega_{MN}^{PUF}$ are the weight fractions of water, o, and MN, respectively, in PUF.

Since PUF contains no solutes other than o and MN, the weight fraction of dry matter in PUF ($\omega_{DM}^{PUF}$) equals

(A15) $$\omega_{DM}^{PUF}=\omega_{o}^{PUF}+\omega_{MN}^{PUF}$$

and summing Equations (A13) and (A14) yields

(A16) $$\left(\omega_{o,dm}^{CD}+\omega_{eMN,dm}^{CD}\right)/\omega_{wdm}^{CD}=\omega_{DM}^{PUF}/\omega_{w}^{PUF}$$

The right side of Equation (A16) was determined from the known value of $\omega_{DM}^{PUF}$:

(A17) $$\omega_{DM}^{PUF}/\omega_{w}^{PUF}={\left({\left(\omega_{DM}^{PUF}\right)}^{-1}-1\right)}^{-1}\equiv a_{DM}$$

where *a_DM_* is a constant for a given PUF.

The right side of Equation (A13) was determined in the same way:

(A18) $$\omega_{o}^{PUF}/\omega_{w}^{PUF}={\left({\left(\omega_{o}^{PUF}\right)}^{-1}-1\right)}^{-1}\equiv a_{o}$$

where *a_o_* is a constant for a given PUF.

Combining Equations (A2), (A13) and (A18), and assuming *f_o_* = 1, *f_SP_* = 0, and *f_eMN_* = 0, yields the following:

(A19) $$\omega_{o,CM}^{CD}=a_{o}\omega_{w,CM}^{CD}$$

The total dry matter content of the *CD* ($\omega_{DM}^{CD}$) was

(A20) $$\omega_{DM}^{CD}=\left(\omega_{mCN}^{CD}+\omega_{aMN}^{CD}+\omega_{o,CM}^{CD}\right)+\left(\omega_{SP}^{CD}+\omega_{o,dm}^{CD}+\omega_{eMN,dm}^{CD}\right)$$

where $\omega_{mCN}^{CD}$, $\omega_{aMN}^{CD}$, and $\omega_{o,CM}^{CD}$ are the weight fractions of the solid components of CMs in the CD (micellar caseins, associated minerals, and absorbed low-molecular-weight organic compounds, respectively); and $\omega_{SP}^{CD}$, $\omega_{o,dm}^{CD}$ and $\omega_{eMN,dm}^{CD}$ are the weight fractions of the solid components of the dm in the CD (SPs, o, and eMN that are not absorbed by CMs, respectively).

Equation (A20) can be rewritten using Equations (2), (3), (A1), (A15)–(A17) and (A19) as

(A21) $$\omega_{DM}^{CD}=\omega_{mCN}^{CD}\left(1+R_{aMN}+a_{o}R_{w}+\frac{R_{SP}}{1-R_{SP}}\right)+a_{DM}\omega_{w,dm}^{CD}$$

where *R_w_* is a parameter defined as

(A22) $$R_{w}=\omega_{w,CM}^{CD}/\omega_{mCN}^{CD}$$

Considering the mass balance

(A23) $$\omega_{w}^{CD}=\omega_{w,CM}^{CD}+\omega_{w,dm}^{CD}$$

and using Equation (A22), the weight fraction of micellar caseins in the compressed dispersion can be calculated from Equation (A21) as

(A24) $$\omega_{mCN}^{CD}=\frac{\left(1+a_{DM}\right)\omega_{DM}^{CD}-a_{DM}}{1+R_{aMN}+\frac{R_{SP}}{1-R_{SP}}+\left(a_{o}-a_{DM}\right)R_{w}}$$

where *R_amn_*, *R_SP_*, and *a_o_* and are the known constants.

For *f_o_* = 1,

(A25) $$\omega_{o,CM}^{CD}/\omega_{w,CM}^{CD}=\omega_{o,dm}^{CD}/\omega_{w,dm}^{CD}=\omega_{o}^{CD}/\omega_{w}^{CD}=a_{o}$$

Thus, parameter *R_w_* can be calculated from Equation (A22) using Equations (A11) and (A25) as

(A26) $$R_{w}=\frac{v_{CM}-\rho_{CN}^{-1}-\rho_{MN}^{-1}R_{aMN}}{\rho_{w}^{-1}+\rho_{o}^{-1}a_{o}}$$

Further, the total composition of the compressed dispersions is calculated as follows:

(a) $\omega_{CN}^{CD}$—from Equation (A24);
(b) $\omega_{SP}^{CD}$—from Equations (2) and (3);
(c) $\omega_{o}^{CD}$—from Equation (A25) using experimentally measured $\omega_{w}^{CD}$ and *a_o_*;
(d) $\omega_{MN}^{CD}$—from Equation (A20)

### Appendix A.5. Composition of WPI Dispersions Prepared via Osmotic Compression

Since the WPI dispersions contained no CMs, the $\omega_{DM}^{CD}$ of the compressed WPI dispersions equaled the sum of $\omega_{SP}^{CD}$, $\omega_{o}^{CD}$, and $\omega_{eMN}^{CD}$. Thus, using the assumptions of Equations (A13) and (A14), along with Equations (A15)–(A17), the weight fractions of the components in the compressed WPI dispersions can be calculated as

(A27) $$\omega_{SP}^{CD}=\omega_{DM}^{CD}-a_{DM}/\left(1+a_{DM}\right)$$

(A28) $$\omega_{o}^{CD}=a_{o}\left(1-\omega_{DM}^{CD}\right)$$

(A29) $$\omega_{MN}^{CD}=\left(a_{DM}-a_{o}\right)\left(1-\omega_{DM}^{CD}\right)$$

### Appendix A.6. Volume Fractions of Components Is Dispersions

The volume fraction of the CMs in dispersions ($\varphi_{CM}^{D}$) was calculated as

(A30) $$\varphi_{CM}^{D}=\rho_{D}v_{CM}\omega_{CN}^{D}$$

where *ρ_D_* is the density of the dispersion calculated from the densities of its components (*ρ_i_*) as

(A31) $$\rho_{D}={\left(\sum_{i}\omega_{i}^{D}/\rho_{i}\right)}^{-1}$$

In Equation (A31), all dispersion components (CNs, SPs, o, MN, and w) were summed regardless of their phase (CMs or dm). The following specific weights (densities) were taken from the literature for calculations: *ρ_CN_* = *ρ_SP_* = *ρ_o_* = 1.35 g·mL^−1^, *ρ_w_* = 1 g·mL^−1^, and *ρ_MN_* = 2.5 g·mL^−1^.

The volume fraction of the SPs in dispersions ($\varphi_{SP}^{D}$) was calculated as

(A32) $$\varphi_{SP}^{D}=\rho_{D}v_{SP}\omega_{SP}^{D}$$

where *v_SP_* is the voluminosity of the SPs.

The total protein volume fraction in the dispersions ($\varphi_{PR}^{D}$) was calculated as

(A33) $$\varphi_{PR}^{D}=\varphi_{CM}^{D}+\varphi_{SP}^{D}$$

and the volume fraction of the SPs in the dm ($\varphi_{SP}^{dm}$) was calculated as

(A34) $$\varphi_{SP}^{dm}=\varphi_{SP}^{D}/\left(1-\varphi_{CM}^{D}\right)$$

regardless of the method used to prepare the dispersion.
