# Supplementary material for: Component Distribution, Shear-Flow Behavior, and Sol–Gel Transition in Mixed Dispersions of Casein Micelles and Serum Proteins
Source: Foods. 2024 Oct 30;13(21):3480. doi: 10.3390/foods13213480 (PMC11545534; doi:10.3390/foods13213480)
Supplement: Supplementary file 1 [file foods-13-03480-s001.zip › foods-3225371-supplementary.pdf]

SUPPLEMENTARY MATERIALS

S1. Comparison of theoretical and measured values of total dry matter content of supernatants obtained from CNI and mixed proteins dispersions

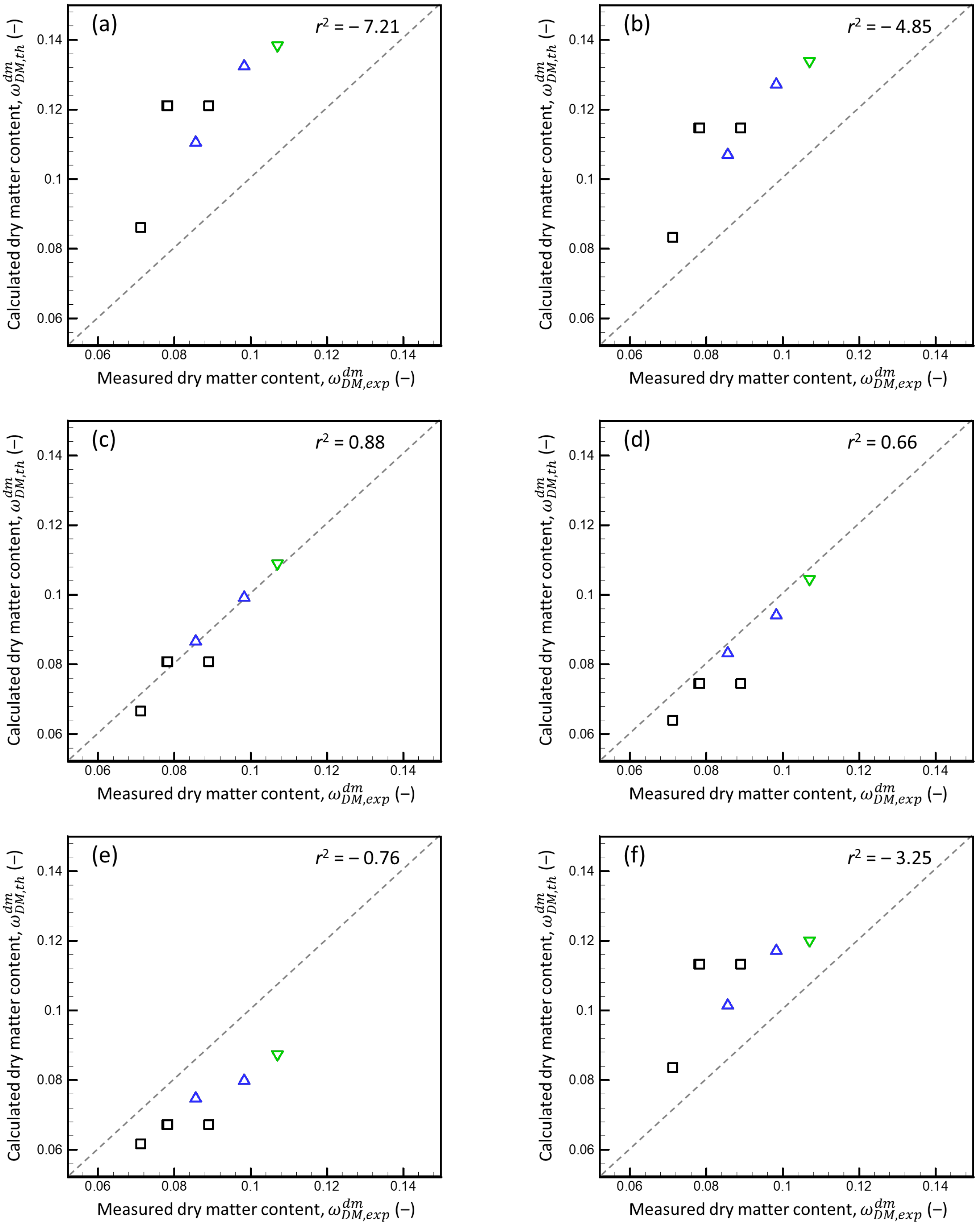

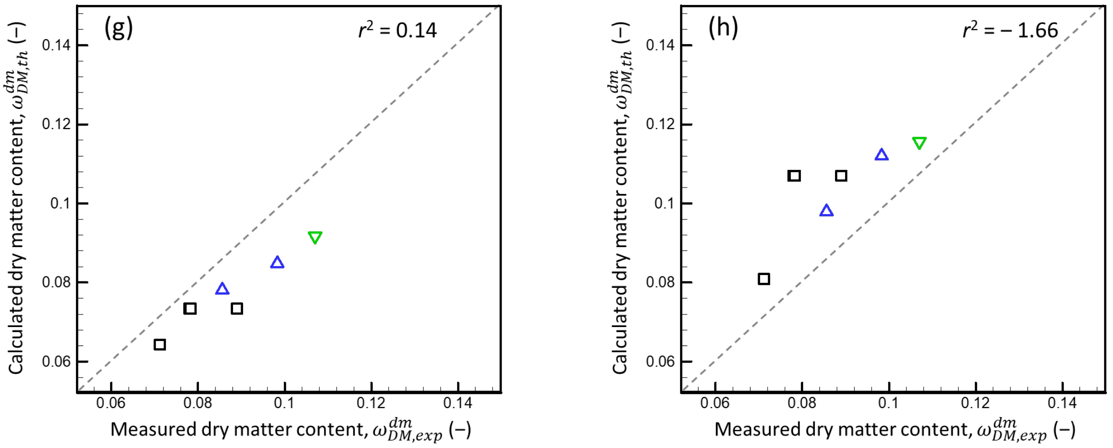

Figure S1. Calculated dry matter content of dispersion medium $\omega_{DM,th}^{dm}$ vs. measured values of total dry matter content of supernatants $\omega_{DM,exp}^{dm}$ of "liquid" mixed milk proteins dispersions. Name of each figure (from (a) to (h)) corresponds to the name of assumption used for the $\omega_{DM,th}$ calculation (Table 2). Data are presented for suspensions with R_{SP} = 0.066 and ω_{PR}^{D} = 0.083 and 0.125 (squares), R_{SP} = 0.158 and ω_{PR}^{D} = 0.105 and 0.126 (triangles), R_{SP} = 0.214 and ω_{PR}^{D} = 0.126 (reverse triangles). Dashed lines correspond to equation $\omega_{DM,exp}^{dm} = \omega_{DM,th}^{dm}$; determination coefficients for the data fitting by this equation (r^2) are shown near the dashed lines. The values on the axes at the origin of coordinates correspond to the dry matter content of PUF: $\omega_{DM,exp}^{dm} = \omega_{DM,th}^{dm} = \omega_{DM}^{PUF}$ = 0.0521.

S2. Values of *K* and *n* for dispersions with various component concentrations

Tables S1 – S4 summarize the data on shear flow behavior of milk protein dispersions: ω_{PR}^{D} – total proteins concentration in dispersion, φ_{CM}^{D} – particle volume fraction of casein micelles in dispersion, φ_{SP}^{dm} – particle volume fraction of SP in dm, *K* and *n* – values of fitting parameters of empirical equation $\tau = K\dot{\gamma}^n$ and the value of determination coefficient r^2 obtained by the fit of experimental data (τ *vs.* $\dot{\gamma}$) in the studied range of shear rates $\dot{\gamma}$ = 1 – 120 or 1 – 1000 (s^{-1}). Volume fractions are calculated for assumed constant values of voluminosities v_{SP} = 2.09 ml·g^{-1} and v_{CM} = 4.15 ml·g^{-1}.

Table S1. Data for CNI dispersions (R_{SP} = 0.066).

Method	ω_{PR}^{D}	φ_{CM}^{D}	φ_{SP}^{dm}	K	n	r^2
Powders	0.021	0.083	0.003	2.17E-03	0.949	1.000
dispersion	0.042	0.166	0.007	2.98E-03	0.945	1.000
	0.051	0.204	0.009	2.51E-03	1.017	1.000
	0.062	0.248	0.012	3.67E-03	0.984	1.000
	0.073	0.292	0.015	4.99E-03	0.965	1.000
	0.083	0.337	0.018	5.92E-03	0.988	1.000
	0.104	0.424	0.026	1.65E-02	0.887	0.999
	0.125	0.512	0.037	7.30E-02	0.815	1.000
Osmotic	0.154	0.632	0.061	5.60E-01	0.662	0.998
compression	0.164	0.679	0.075	2.20E+01	0.284	0.983
	0.173	0.716	0.089	2.87E+01	0.261	0.981

Table S2. Data for mixed protein dispersions with R_{SP} = 0.158.

Method	ω_{PR}^{D}	φ_{CM}^{D}	φ_{SP}^{dm}	K	n	r^2
Powders	0.021	0.075	0.008	1.62E-03	0.999	1.000
dispersion	0.042	0.151	0.017	1.73E-03	1.005	1.000
	0.063	0.227	0.028	3.48E-03	0.982	1.000
	0.067	0.243	0.030	4.56E-03	0.930	1.000
	0.084	0.305	0.041	5.31E-03	0.969	1.000
	0.090	0.330	0.047	6.87E-03	0.949	1.000
	0.102	0.374	0.057	8.11E-03	0.969	1.000
	0.126	0.464	0.082	2.33E-02	0.928	1.000
Osmotic	0.165	0.615	0.151	1.63E+00	0.551	0.999
compression	0.182	0.681	0.202	2.29E+01	0.303	0.986
	0.191	0.715	0.237	5.55E+01	0.253	0.984

Table S3. Data for mixed protein dispersions with R_{SP} = 0.214.

Method	ω_{PR}^{D}	φ_{CM}^{D}	φ_{SP}^{dm}	K	n	r^2
Powders	0.021	0.070	0.010	1.80E-03	0.964	1.000
dispersion	0.042	0.141	0.023	2.19E-03	0.992	1.000
	0.063	0.213	0.037	3.20E-03	0.982	1.000
	0.084	0.285	0.055	5.18E-03	0.966	1.000
	0.105	0.359	0.077	8.05E-03	0.975	1.000
	0.126	0.434	0.105	1.50E-02	0.969	1.000
Osmotic	0.157	0.545	0.164	8.16E-02	0.816	1.000
compression	0.160	0.556	0.172	2.65E-01	0.746	0.999
	0.175	0.608	0.214	3.61E+00	0.470	0.999
	0.180	0.628	0.232	8.97E+00	0.409	0.997
	0.193	0.675	0.285	3.10E+01	0.246	0.937
	0.203	0.711	0.338	9.55E+01	0.121	0.662

Table S4. Data for WPI dispersions (R_{SP} = 1).

Method	ω_{PR}^{D}	φ_{CM}^{D}	φ_{SP}^{D}	K	n	r^2
Powders	0.022	0	0.047	1.80E-03	0.963	1.000
dispersion	0.044	0	0.094	2.22E-03	0.966	1.000
	0.066	0	0.142	2.84E-03	0.968	1.000
	0.087	0	0.190	2.90E-03	0.995	1.000
	0.087	0	0.190	2.57E-03	0.988	1.000
	0.109	0	0.239	4.10E-03	0.949	1.000
	0.131	0	0.289	4.59E-03	0.981	1.000
	0.131	0	0.289	4.00E-03	0.994	1.000
	0.140	0	0.309	4.18E-03	1.007	1.000
	0.153	0	0.339	5.52E-03	0.998	1.000
	0.166	0	0.369	6.20E-03	0.993	1.000
	0.175	0	0.390	7.30E-03	0.985	1.000
Osmotic	0.162	0	0.362	6.98E-03	0.932	0.999
compression	0.194	0	0.436	9.73E-03	0.945	1.000
	0.223	0	0.507	2.70E-02	0.933	0.999
	0.286	0	0.664	1.28E-01	0.922	0.999

S3. Relative viscosity of WPI dispersions in PUF at various shear rates described by equation of Quemada

The equation of Quemada for the concentration dependency of the relative viscosity of a dispersion of hard spheres is

$$\eta_r = (1 - \varphi/\varphi_m)^{-2} \quad \text{(S1)}$$

where φ_m is the volume fraction of particles in the system at which the viscosity of the system diverges ($\eta_r \to \infty$ for $\varphi \to \varphi_m$, when the particles in the system behave as hard spheres) [9]. In Figure S2, the dependencies of relative viscosity of WPI dispersions η_{rPUF} on volume fraction of SP φ_{SP}^D are presented in the coordinates of the linearized form of Equation (S1), $\eta_r^{-1/2}$ *vs.* φ

$$\eta_r^{-1/2} = 1 - \varphi/\varphi_m \quad \text{(S2)}$$

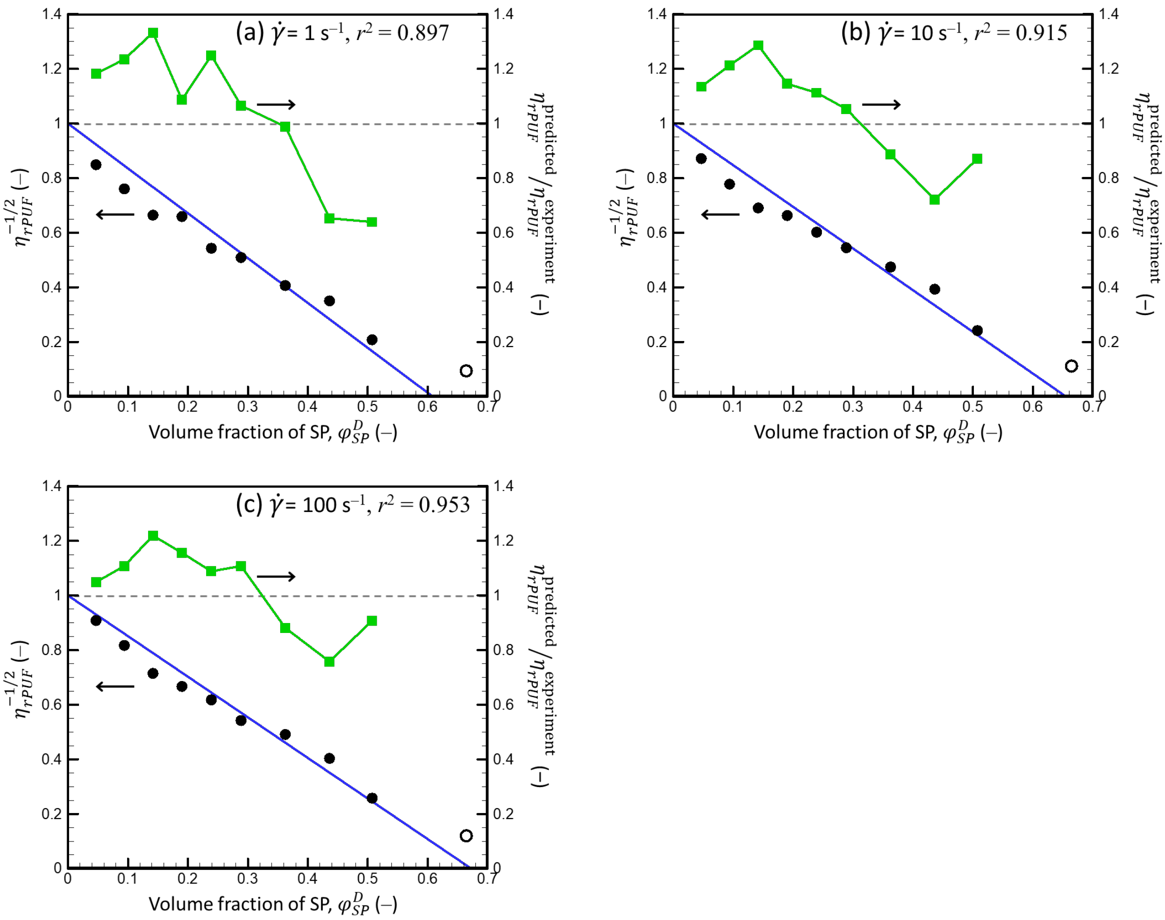

Figure S2. Left axis – function of relative viscosity of WPI dispersions *vs.* φ_{SP}^D (symbols – experimental data, solid symbols – data used for the fit by Equation (S2), solid lines – obtained by data fit with Equation (S2)); determination coefficient for each fit (r^2) is shown on each figure.

Right axis – relative viscosity obtained from Equation (S1) divided by the value of η_{rPUF} obtained in experiment *vs.* φ_{SP}^{D}, dotted horizontal corresponds to $\eta_{rPUF}^{predicted}/\eta_{rPUF}^{experiment} = 1$.

Data for $\dot{\gamma}$ = 1 s^{-1} (a), 10 s^{-1} (b), and 100 s^{-1} (c).

It can be concluded that regardless of $\dot{\gamma}$, the dependencies of $\eta_r^{-1/2}$ on φ_{SP}^{D} were non-linear in the range φ_{SP}^{D} = 0 – 0.55, and thus cannot be described adequately by Equation (S1) when a single value of the fitting parameter φ_m is used for a given $\dot{\gamma}$. It is known that the value of the maximal packing volume fraction of particles, φ_m in Equation (S1), can increase with the increase of particles volume fraction in dispersion [34]. However, the dependency of φ_m on φ is generally unknown, which makes the application of Eq (S1) for the description of obtained dependencies $\eta_r^{-1/2} = f(\varphi_{SP}^{D})$ non-practical.

S4. Relative viscosity of WPI dispersions in PUF at various shear rates described by the equation of Mendoza–Santamaria-Holek

Mendoza and Santamaria-Holek [16] debated the validity of Equation (S1) and proposed an improved equation for relative viscosity:

$$\eta_r = (1 - (1 + \varphi^{-1} - \varphi_m^{-1})^{-1})^{-5/2} \quad \text{(S3)}$$

where the parameter φ_m has the same meaning as in Equation (S1).

In Figure S3, the dependencies of relative viscosity of WPI dispersions η_{rPUF} on volume fraction of SP φ_{SP}^{D} are presented in the coordinates of the linearized form of Equation (S3)

$$\frac{1}{1-\eta_{rPUF}^{-2/5}} - 1 = \varphi^{-1} - \varphi_m^{-1} \quad \text{(S4)}$$

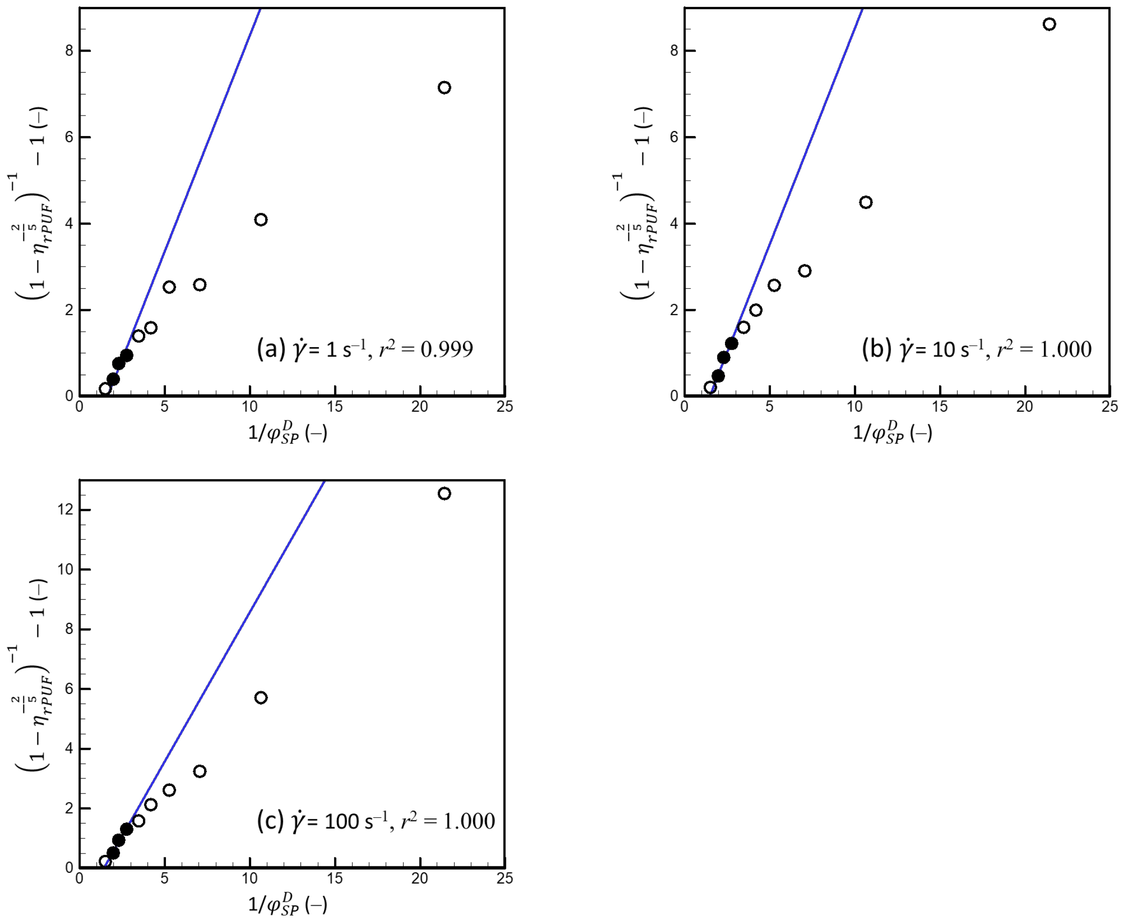

Figure S3. Function of relative viscosity of WPI dispersions *vs.* $1/\varphi_{SP}^{D}$ (symbols – experimental data, solid symbols – data used for the fit by Equation (S4), solid lines – obtained by data fit with Equation (S4)); determination coefficient for each fit (r^2) is shown on each figure. Data for $\dot{\gamma}$ = 1 s^{-1} (a), 10 s^{-1} (b), and 100 s^{-1} (c).

As well as Equation (S2), Equation (S4) did not describe the data on relative viscosity of WPI dispersions in the entire range of φ_{SP}^{D}. It is worth to noting that, in the region of low φ (high $1/\varphi$), even small deviation of the experimentally measured η_r from that predicted by the model results in a very large deviation of the point [φ; η_r] from the model curve, when the data are presented in the linearized coordinates of Equation (S4). In any case, regardless of the data presentation, the use of points experimentally measured points with $\eta_r \rightarrow 1$ is only practical when the data are obtained with very low experimental error, because $\eta_r \rightarrow 1$ when $\varphi \rightarrow 0$ in the frame of any rheological model. Nevertheless, an excellent fit was obtained for the concentration range $\varphi_{SP}^{D} \approx 0.36 - 0.51$. Interesting, that Equation (S1) is also suitable for the description of viscosity in this concentration range (Figure S2).

S5. Relative viscosity of mixed proteins dispersions described by equations of Quemada and Mendoza–Santamaria-Holek

In Figures S4 and S5, the dependencies of relative viscosity of dispersions η_r on volume fraction of casein micelles in mixed dispersions φ_{CM}^{D} are presented in the coordinates of the linearized form of the equation of Quemada, Equation (S2), and Mendoza–Santamaria-Holek, Equation (S4), respectively.

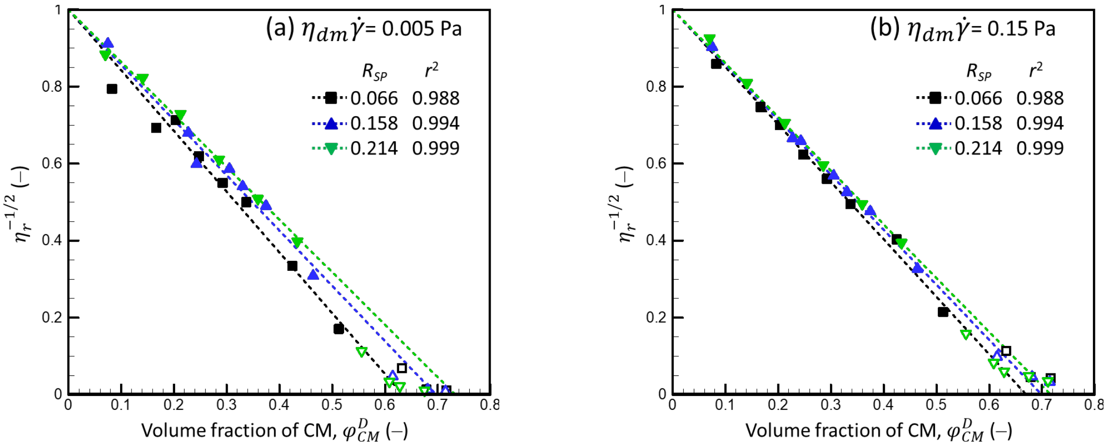

Figure S4. Function of relative viscosity of mixed proteins dispersion calculated for $\eta_{dm}\dot{\gamma}$ = 0.005 Pa (a) and 0.15 (b) *vs.* casein micelle volume fraction in dispersion φ_{CM}^{D} for dispersions with R_{SP} = 0.066 (squares), 0.158 (triangles), 0.214 (reverse triangles): symbols – experimental data; solid symbols – data used for the data fit with Equation (S2); dashed lines – calculated *via* fit with Equation (S2). Determination coefficient for each fit (r^2) is shown near symbols.

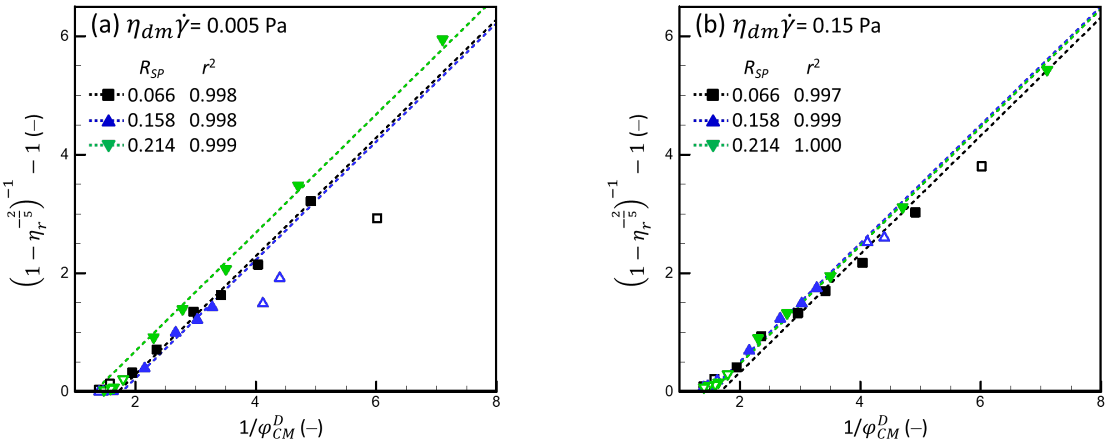

Figure S5. Function of relative viscosity of mixed protein dispersion calculated for $\eta_{dm}\dot{\gamma}$ = 0.005 Pa (a) and 0.15 (b) *vs.* casein micelle volume fraction in dispersion φ_{CM}^D for dispersions with R_{SP} = 0.066 (squares), 0.158 (triangles), 0.214 (reverse triangles): symbols – experimental data; solid symbols – data used for the data fit with Equation (S4); dashed lines – calculated *via* fit with Equation (S4). Determination coefficient for each fit (r^2) is shown near symbols.

Both equations satisfactorily fit the experimental dependencies up to $\varphi_{CM}^D \approx 0.5$ with comparable values of r^2 (as it is explained above, the data obtained at low φ_{CM}^D are not considered for the fit with Equation (S4)). Regardless of $\eta_{dm}\dot{\gamma}$, at higher φ_{CM}^D, Equation (S2) underestimates the relative viscosity for R_{SP} = 0.158 and 0.214. This can be explained by the increase of viscosity due to increased repulsions between the CM in the presence of SP, which is not considered by the model of Quemada. In Figure 7, this behavior is equivalent to steep increase of viscosity with R_{SP} for $\varphi_{CM}^D > 0.55$. At $\varphi_{CM}^D > 0.7$, i.e. after the point of sol-gel transition (Figure 10), Equation (S2) overestimates the viscosity for all studied dispersions, since the model does not consider the flow of the "caged" particles due to their deformation under the applied shear stress. In Figure 7, this behavior is equivalent to a lesser dependence of viscosity on R_{SP} in most concentrated dispersions. The observations for the data fit with Equation (S4) (shown in Figure S5) are basically the same as for that with Equation (S2).

Expectedly, the data fit with Eqs. (S2) and (S4) yields different values of parameter φ_m (Figure S6): except for a few points, the values φ_m obtained with the help of Equation (S4) are significantly lower than those obtained with Equation (S2) because of the difference in the way the free volume is accounted for in the models of Quemada and Mendoza–Santamaria-Holek. Nevertheless, both models predict the value of the maximal φ_{CM}, where the system is jammed,

in the vicinity of the concentration of sol-gel transition found from the oscillatory experiments (≈ 0.65).

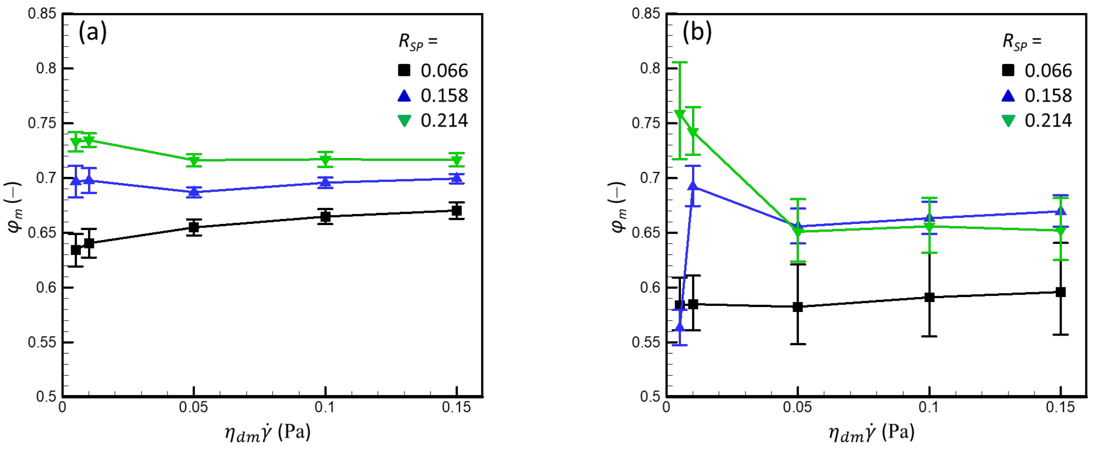

Figure S6. Values of fitting parameter φ_m obtained with the help of Eqs. (S2) (a) and (S2) (b) *vs.* $\eta_{dm}\dot{\gamma}$. Error bars correspond to 95% confidential interval of the fit. Data for dispersions with R_{SP} = 0.066 (squares), 0.158 (triangles), 0.214 (reverse triangles).

S6. Schematic diagram of the preparation of milk protein dispersions

The method used for the preparation of diluted and compressed dispersions of WPI, CNI as well as of their mixtures (which is explained in detail in Materials and methods) is schematically presented in Figure S7.

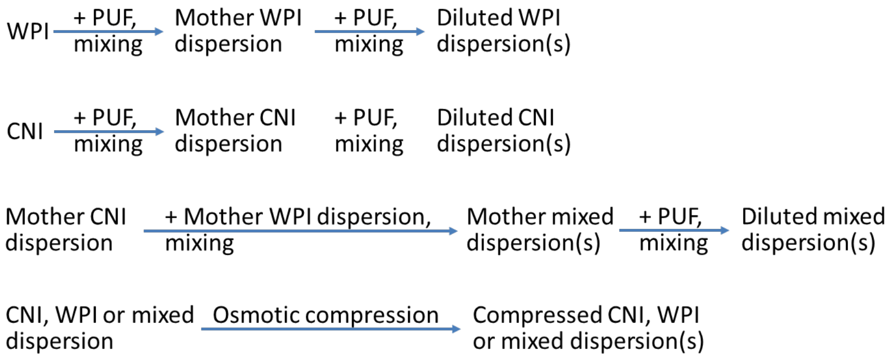

Figure S7. Schematic diagram of the preparation of milk protein dispersions.

In Figure S7, WPI corresponds to whey protein isolate powder, CNI – native phosphocaseinate isolate powder, PUF – permeate of skim milk ultrafiltration. We acknowledge Reviewer 2, who recommended presenting this diagram.
